# Identification of Novel *Clostridium perfringens* Type E Strains That Carry an Iota Toxin Plasmid with a Functional Enterotoxin Gene

**DOI:** 10.1371/journal.pone.0020376

**Published:** 2011-05-31

**Authors:** Kazuaki Miyamoto, Natsuko Yumine, Kanako Mimura, Masahiro Nagahama, Jihong Li, Bruce A. McClane, Shigeru Akimoto

**Affiliations:** 1 Department of Microbiology, Wakayama Medical University School of Medicine, Wakayama, Japan; 2 Department of Microbiology, Faculty of Pharmaceutical Sciences, Tokushima Bunri University, Tokushima, Japan; 3 Department of Microbiology and Molecular Genetics, University of Pittsburgh School of Medicine, Pittsburgh, Pennsylvania, United States of America; The University of Hong Kong, Hong Kong

## Abstract

*Clostridium perfringens* enterotoxin (CPE) is a major virulence factor for human gastrointestinal diseases, such as food poisoning and antibiotic associated diarrhea. The CPE-encoding gene (*cpe*) can be chromosomal or plasmid-borne. Recent development of conventional PCR *cpe*-genotyping assays makes it possible to identify *cpe* location (chromosomal or plasmid) in type A isolates. Initial studies for developing *cpe* genotyping assays indicated that all *cpe*-positive strains isolated from sickened patients were typable by *cpe*-genotypes, but surveys of *C. perfringens* environmental strains or strains from feces of healthy people suggested that this assay might not be useful for some *cpe*-carrying type A isolates. In the current study, a pulsed-field gel electrophoresis Southern blot assay showed that four *cpe*-genotype untypable isolates carried their *cpe* gene on a plasmid of ∼65 kb. Complete sequence analysis of the ∼65 kb variant *cpe*-carrying plasmid revealed no intact IS elements and a disrupted cytosine methyltransferase (*dcm*) gene. More importantly, this plasmid contains a conjugative transfer region, a variant *cpe* gene and variant iota toxin genes. The toxin genes encoded by this plasmid are expressed based upon the results of RT-PCR assays. The ∼65 kb plasmid is closely related to the pCPF4969 *cpe* plasmid of type A isolates. MLST analyses indicated these isolates belong to a unique cluster of *C. perfringens*. Overall, these isolates carrying a variant functional *cpe* gene and iota toxin genes represent unique type E strains.

## Introduction

*Clostridium perfringens*, a gram-positive spore-forming anaerobic bacterium, has a ubiquitous presence in the normal intestinal flora, feces, soil and sewage [1], [2], [3]. This bacterium produces many different toxins, at least 16 [1]. Based upon their production of four typing toxins (alpha, beta, epsilon, and iota), *C. perfringens* isolates are classified into five toxin types (A to E).

*C. perfringens* type A is one of the most common causative agents of human gastrointestinal (GI) diseases, such as food poisoning, antibiotic associated diarrhea, and sporadic diarrhea [1]. *C. perfringens* enterotoxin (CPE) is the most important virulence factor when type A isolates cause human GI diseases, although only ∼5% of type A isolates produce this toxin [1], [4]. Recent studies indicated that *C. perfringens* type A isolates causing food poisoning or human GI disease classify into three groups based upon their enterotoxin gene (*cpe*) locus [5]. These groups include chromosomal *cpe* isolates (carrying a *cpe*-IS*1470* locus) and two plasmid-borne *cpe* groups that carry either a *cpe*-IS*1151* locus or a *cpe*-IS*1470*-like locus [5].

Using a PCR assay capable of distinguishing amongst those three *cpe* loci, surveys identified several *cpe*-positive isolates from the feces of healthy humans or the environment that possess an as yet uncharacterized *cpe* locus arrangement [2], [3], [6]. The significance of these isolates has been unclear, but some are cytotoxic and might produce CPE [2], [6]. So, a further investigation of these isolates carrying an uncharacterized *cpe* locus might shed light on their potential clinical significance and on the evolution of the *cpe* gene itself.

Therefore this study conducted a genotypic and phenotypic characterization of *C. perfringens* isolates with an uncharacterized *cpe* locus. This work included analyzing in these isolates: *cpe* sequence diversity, the *cpe* location (chromosome or plasmid), toxin expression and their genetic background.

## Results

### Properties of *cpe*-positive isolates with an unknown *cpe* locus arrangement

The current study examined four *cpe*-positve isolates, PB-1, 3441, TGII002, and TGII003. When assayed by a multiplex toxinotyping PCR, these four *cpe*-positive isolates all tested as *cpe*-positive type A *C. perfringens* [8]. However, these isolates each carried an unknown *cpe* locus arrangement, as tested using a *cpe* genotyping assay [5].

Analysis of the *cpe* gene sequence in PB-1, 3441, TGII002, and TGII003 revealed that each of these isolates carries an identical variant *cpe* gene, with the same sequence as that found in USA soil isolate S292-3, which may produce undetectable levels of CPE and has an unusual organization of its *cpe* promotor region sequence [2]. Comparing this variant *cpe* gene against the classical *cpe* gene found in isolates with a known *cpe* locus arrangement, the two *cpe* genes were highly homologous at the amino acid level (identities  = 307/319 (96%), positives  = 315/319 (98%)). Of ten amino acid differences, only one amino acid change was found in the cell binding region of CPE and no amino acid changes were found in the major cytotoxicity region (Figure S1). With respect to nucleotide diversity, comparison of the variant vs. classical *cpe* genes revealed 33 nucleotide differences.

**Figure 1:**
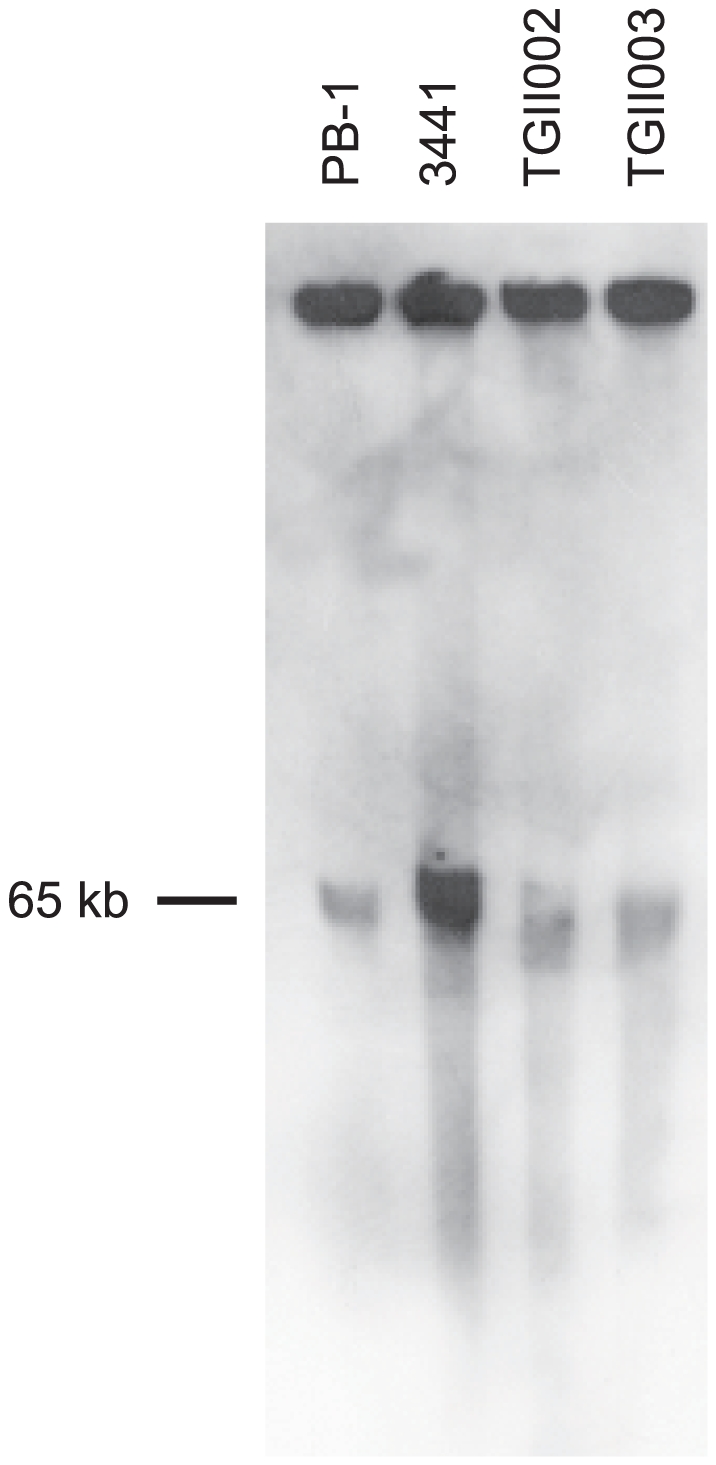
PFGE *cpe* Southern blot analysis of DNA from strains carrying the atypical *cpe* gene. A. Four isolates (PB-1, 3441, TGII002, and TGII003) carrying the variant *cpe* locus carried an ∼65 kb *cpe* plasmid.

Southern blot analysis of pulsed-field gels showed that all four surveyed isolates with an unusual *cpe* locus arrangement carry their *cpe* gene on an ∼65 kb plasmid (Figure 1).

### Complete sequence analysis of the ∼65 kb variant *cpe*-encoding plasmid, pCPPB-1

**Figure 2:**
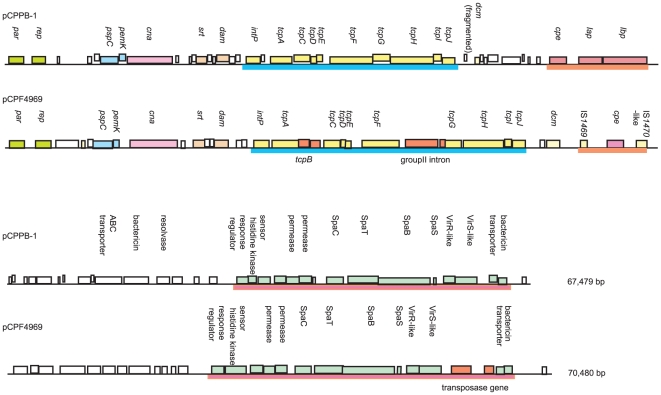
Comparative alignment of pCPPB-1 variant *cpe* plasmid versus pCPF4969 classical *cpe*-plasmid. Map displays ORFs identified by ORF Finder using the obtained the variant *cpe*-encoding plasmid (pCPPB-1) sequence. A blue line marks the putative plasmid transfer region, an orange line delineates toxin, and a pink line indicated the region homologous with a part of pCPF4969 variable region. Potential roles of each ORF are indicated by the key in the lower right-hand corner of the figure.

Complete sequencing of the *cpe* plasmid (named pCPPB-1) in PB-1 revealed a size of 67,479 bp, encoding 72 putative ORFs (Figure 2). Analysis of pCPPB-1 revealed that this plasmid consists of three regions: a putative plasmid replication and transfer region, a toxin region, and a variable region.

**Figure 3:**
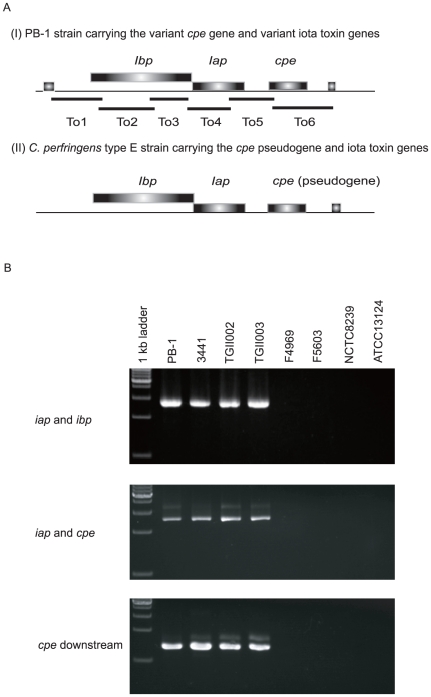
Genetic organization of the variant *cpe* toxin locus. **A.** Genetic organization of the toxin locus in pCPPB-1 versus the toxin locus in previously studied *C. perfringens* type E strain (JGS1987) [16]. Broad bars show ORFs. Long thin bars depict the PCR products as shown in Panel B, amplified with each primer pair (see the text). **B.** PCR analysis of the toxin locus in *C. perfringens* isolates carrying the variant *cpe* gene.

Surprisingly, this sequencing detected the presence of two ORFs encoding homologues of the iota toxin genes that characterize *C. perfringens* type E strains. These iota toxin genes of PB-1 strain resided near the variant *cpe* gene (Figure 3A). The toxin locus in pCPPB-1 has a generally similar organization as the iota toxin plasmid of classical type E strains, except the *cpe* gene is intact and not disrupted by the nonsense and frame-shift mutations found in other type E strains (Figure 3A) [16].

Concerning the iota toxin genes of pCPPB-1, analysis of the *iap* ORF indicated that the iA component encoded by this plasmid is highly homologous with the consensus iA component of type E iota toxin and the ADP-ribosylation component of the CDT toxin made by *Clostridium difficile* (Figure S2). The most different region between the iA component encoded by pCPPB-1 vs. the classical iA is the signal peptide region. The pCPPB-1 putative signal peptide for iA should be functional, based on analysis of the signal peptide sequence by SOSUI software (http://bp.nuap.nagoya-u.ac.jp/sosui/) [17]. The iB component encoded by pCPPB-1 is also homologous with the iB component of consensus iota toxin (Figure S3).

The nucleotide sequence of the variant *iap* gene on pCPPB-1 shared 91% (1252/1376) identity with 1% (22/1376) gaps, and 87% (1194/1376) identity with 2% (36/1376) gaps, compared against the consensus *iap* gene of previously examined *C. perfringens* type E strain. Interestingly, many nucleotide differences are also present in the first ∼750 bp on 5′ site, a location containing the sites for hybridization of the *iap* primers commonly used in the toxin genotyping assay based on the *iap* gene sequence in previously investigated type E isolates. In fact, five of twenty nucleotides on the forward *iap* primer and two of 21 nucleotides in the reverse primer differ in the variant *iap* gene present on pCPPB-1 [8] (Figure S4).

The *ibp* gene of pCPPB-1 also shared high identities with the *ibp* gene in *C. perfringens* type E strains, (89% identities with 2% gaps) and the *cdtB* gene in *C. difficile* (85% identities with 4% gaps) (Figure S5). Nucleotide differences were broadly distributed between the variant *ibp* gene on pCPPB-1 and the consensus *ibp* gene in previously studied type E strains, although the 3′ region of the gene was highly homologous with the consensus *ibp* gene of previously studied type E strains (Figure S5).

A previous study suggested that the upstream region of the same variant *cpe* gene present in soil isolate S292-3 might have a defective promoter region [2]. Sequencing of the *cpe* promoter region on pCPPB-1 showed that this *cpe* gene had a putative ribosome binding site and putative SigK-dependent and putative SigE-dependent promoters (P1 and P2, respectively), but lacked the most upstream SigE-depedent promoter, P3 [18], [19] (Figure S6).

**Figure 4:**
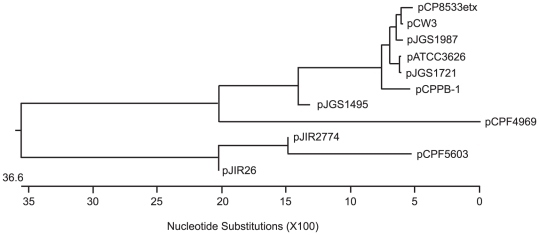
Phylogenetic relationships of the *tcpA* gene among toxin plasmids and antibiotics resistant gene encoding plasmids of *C. perfringens*. The phylogenetic tree was constructed by Clustal W analysis based on the nucleotide sequence of *tcpA* genes (accession numbers; pCPF4969, NC007772; pCPF5603, NC007773; pCP8533etx, AB444205; pCW3, DQ366035; pJGS1987, NZ_ABDW01000012; pATCC3626, ABDV01000020; pJGS1721, NZ_ABOO01000017; pJGS1945, NZ_ABDU01000064; pJIR2774, DQ338473; and pJIR26, DQ338471).

The putative plasmid replication and conjugative transfer region of pCPPB-1 contains *tcp* genes (*intP*, *tcpA*, *tcpC* to *tcpJ*), putative adenine methyltransfease gene (*dam*), and collagen adhesion protein gene (*cna*), but not a putative intact cytosine methyltransfease gene (*dcm*). To investigate the genetic relationship of the pCPPB-1 plasmid with other toxin plasmids, the *tcpA* gene sequence in PB-1 was compared against the *tcpA* gene on other toxin plasmids, with the rationale that the TcpA protein is the most divergent of the transfer-related proteins carried on conjugative transferable *C. perfringens* plasmids [20]. Based on the sequence information obtained for the *tcpA* gene, pCPPB-1 appears to be more closely related with the iota toxin plasmid in type E strain rather than the type A *cpe* plasmids (Figure 4).

### PCR-based diversity analysis of the isolates carrying the variant *cpe* plasmid

The four isolates with the variant *cpe* gene carried five representative genes found on the prototype classical *cpe* plasmids pCPF5603 and pCPF4969; three of these are essential conjugative transfer genes (*tcpA*, *tcpF*, and *tcpH*), another is a putative collagen adhesion gene (*cna*), and the last a putative adenine-specific methyltransferase gene (*dam*) (Table 1) [12]. Moreover, overlapping PCR assays for the transfer region suggested that the *cpe* plasmid in PB-1, 3441, TGII002, and TGII003 might carry the transfer region (*intP*, *tcpA* to *tcpI* genes) homologous with other conjugative toxin plasmids in *C. perfringens* (Figure S7). However, the putative cytosine-specific methyltransferase gene (*dcm*), commonly found on other *cpe* plasmids was not detected in any of the four variant *cpe* isolates. Nor did these isolates carry any of the three *cpe*-related IS elements (IS*1151*, IS*1469* and IS*1470*), commonly found on pCPF5603, pCPF4969 and some other type A to E toxin plasmids (Table 1) [12], [13], [21]–[24].

**Table 1:** PCR survey of 18 genes found on toxin-encoding plasmids in *C. perfringens*.

	type	*cpb2*	*tcpA*	*tcpF*	*tcpH*	ETX-*rep*	CPE *rep*	*cna*	*dcm*	*dam*	IS *1469*	IS *1151*	IS *1470*	*capK*	*comEC*	*thiF*	*spaC*	*virS*	*soj*	*parB*	Reference
PB-1	E	-	+	+	+	+	-	+	-	+	-	-	-	-	-	-	+	+	-	-	This study
3441	E	-	+	+	+	+	-	+	-	+	-	-	-	-	-	-	+	+	-	-	This study
TGII002	E	-	+	+	+	+	-	+	-	-	-	-	-	-	-	-	+	+	-	-	This study
TGII003	E	-	+	+	+	+	-	+	-	+	-	-	-	-	-	-	+	+	+	-	This study
S292-3	A	-	+	+	+	NT	NT	+	-	+	+	+	NT	+	+	-	NT	NT	NT	NT	2
S350-1	A	-	+	+	+	NT	NT	+	+	+	+	+	NT	-	-	+	NT	NT	NT	NT	2
NCTC8084	E	+	+	+	+	+	+	+	+	+	-	+	+	-	-	-	-	-	+	-	This study
NCIB10748	E	+	+	+	+	NT	NT	+	+	+	NT	+	NT	+	-	-	NT	NT	NT	NT	13
JGS1987	E	-	+	+	+	+	-	+	+	+	-	+	-	+	+	+	-	-	-	-	13
F5603	A	+	-	+	+	+	+	+	+	+	+	+	NT	+	+	+	NT	NT	+	+	9, This study
F4969	A	-	-	+	+	NT	+	+	+	+	+	NT	-	NT	NT	NT	+	+	NT	NT	9, This study
NCTC8533	B	+	+	+	+	+	+	+	+	+	NT	+	NT	NT	NT	NT	NT	NT	+	+	9, This study
NCTC8081	C	-	-	+	-	+	+	+	+	+	NT	NT	+	NT	NT	NT	NT	NT	+	+	9, This study
ATCC13124	A	-	-	-	-	-	-	-	-	-	-	-	-	-	-	-	-	-	-	-	9, This study

The first four isolates in this table are unique group of type E isolates.

NT: not tested.

The classical iota toxin plasmid found in type E isolates is related to pCPF5603 [13]. However, pCPPB-1 is more related to the *cpe*-plasmid pCPF4969, based on comparative sequence alignment (Figure 2). Furthermore, overlapping PCR results for the pCPF4969 variable region suggest that the *cpe* plasmid in 3441, TGII002, and TGII003 is also related to pCPF4969 (Figure S8). Despite this similarity, the putative transfer region of pCPPB-1 lacked the *tcpB* gene and the group II intron sequence found on pCPF4969 [11]. Moreover, pCPPB-1 lacks a *dcm* gene and IS elements characteristic of other *C. perfringens* toxin plasmids.

The pCPPB-1 plasmid toxin region contained variant *cpe*, *iap*, and *ibp* genes. PCR assays for the PB-1 toxin locus indicated that the three other investigated variant *cpe* isolates carried a similar toxin locus (Figure 3B). Conservation of the toxin locus in the four isolates carrying the variant *cpe* gene was confirmed by overlapping PCR assays (Figure S9).

### MLST analysis of the isolates carrying the variant *cpe* genes

**Figure 5:**
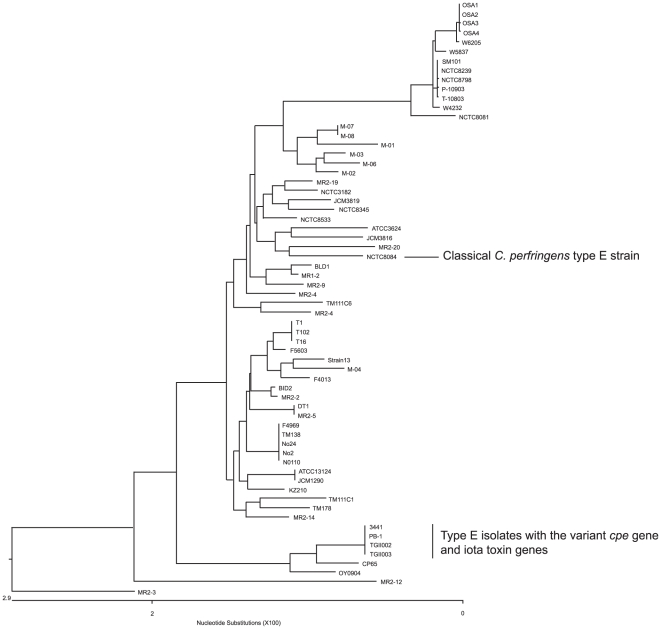
Phylogenetic relationships among *cpe*-positive or *cpe*-negative *C. perfringens* strains containing *C. perfringens* type B to E strains. The phylogenetic tree was constructed by Clustal W analysis based on the concatenated nucleotide sequence of eight housekeeping genes as described previously [8].

Since pCPPB-like plasmids are a new family of iota toxin plasmid, we investigated the genetic relationship of the surveyed isolates carrying this new iota toxin plasmid against other isolates. This involved, a genetic analysis of their chromosomal house-keeping genes using a MLST assay [9]. Interestingly, all four isolates of these newly identified type E isolates localized to a distinct cluster, which was different from type B to E animal isolates (Figure 5).

### RT-PCR analysis of toxin gene transcription

The sequence information obtained for pCPPB-1, along with the results of toxin gene PCR assays, suggested that the four surveyed isolates might carry functional toxin genes. To investigate if those genes are transcribed, RT-PCR assays were performed using RNA preparations from TGY cultures or Duncan/Strong cultures. The four surveyed isolates transcribed *cpe* mRNA during sporulation (Table 2), strongly suggesting these isolates are enterotoxigenic, particularly since their CPE amino acid sequences are highly conserved relative to the classical CPE that is an enterotoxin.

**Table 2:** RT-PCR assays of the toxin genes on pCPPB-1.

	*ia*	*ib*	*cpe*	*sigK*
	RT-PCR	RT-PCR	RT-PCR	RT-PCR
PB-1	+	+	+	+
3441	+	+	+	+
TGII002	+	+	+	+
TGII003	+	+	+	+
NCTC8239	NT	NT	+	+

NT: not tested.

RT-PCR analyses also detected transcription of the iota toxin genes in these four isolates. This suggests that these four isolates produce active iota toxin (Ia and Ib), particularly given the conserved nature of the amino substitutions of these components, as described above (Table 2).

### Sequence analysis of the *ssp4* gene in the four type E isolates carrying a variant *cpe* gene

Sequencing of the *ssp4* gene in the four type E isolates carrying variant *cpe* and iota toxin genes revealed that the small soluble acid protein (SASP) Ssp4 in these isolates has Gly at residue 36 and Lys at residue 72. This contrasts with the *ssp4* gene of type A chromosomal *cpe* strains, which form spores that are highly resistant to stresses such as heat. The *ssp4* gene of those isolates encodes an Asp at residue 36, while residue 72 encodes an Asn [14], [15]. These findings for the four isolates carrying the variant *cpe* gene suggested that they form relatively heat-sensitive spores. This was confirmed by demonstrating that the D value at 100°C for spores from PB-1 and strain 3441 is <2 min (data not shown), in contrast to the D_100_ value of chromosomal *cpe* strains forming heat-resistant spores, which averages more than 15 min [7], [14].

## Discussion

The current study first identified a variant *cpe* gene, which was encoded on an ∼65 kb plasmid. While variants of other *C. perfringens* toxins have been reported, this finding marks (to our knowledge) the first identification of isolates that likely produce a variant CPE. Supporting this possibility, the variant *cpe* gene can be transcribed during sporulation which, given its sequence similarity to the classical *cpe* gene, should lead to production of a functional CPE protein. This should be investigated in the future.

The isolates carrying this variant *cpe* gene initially appeared to be type A isolates based upon initial results using the standard multiplex PCR toxinotyping assay. Surprisingly, *cpe* plasmid sequencing and PCR analyses identified these isolates as type E, since variant iota toxin genes were detected nearby the *cpe* gene. RT-PCR analyses indicated that these variant iota toxin genes are transcribed. Thus, it is possible that CPE and iota toxin might act together if these isolates are pathogenic. Further surveys should assess whether these novel type E isolates can cause disease in animal models and whether they can be isolated from naturally ill people or animals.

The discovery of the variant iota toxin genes on pCPPB-1-like plasmids is the most important finding of the current study since it reveals the existence of, at least, two families of iota toxin plasmids. The previously known iota toxin plasmids are variable in size but have a pCPF5603 backbone, IS*1151* sequences in their toxin locus, a lambda toxin gene, and carry a nonfunctional *cpe* gene that is silent due to mutations in the promoter and ORF. In contrast, the newly identified (this study) pCPPB-1 iota toxin plasmid family is a consistent ∼65 kb size, has a pCPF4969 backbone with no IS elements located near its toxin locus, lacks a lambda toxin gene, and carries a functional *cpe* gene. However, both iota toxin plasmid families are likely to be conjugative based upon their carriage of *tcp* genes [20].

There is emerging evidence that many, but not all, *C. perfringens* toxin plasmids are related to pCPF5603 or pCPF4969. The two type E iota toxin plasmid families clearly resemble either pCPF5603 or pCPF4969, as already discussed. Previous studies have also shown that the epsilon toxin plasmid of type B isolates, and a few type D isolates, also resembles pCPF5603 [10]. More recently, the NetB toxin-encoding plasmid was reported to share similarity with pCPF5603 [25]. Since most toxin genes are closely associated with IS sequences, it is thought that mobile genetic elements carrying various toxin genes have inserted into plasmid backbones common to many *C. perfringens* strains. Therefore, it is notable that IS sequences are not present in the toxin locus of pCPPB-1, possibly because those IS sequences were lost during excision or insertion of the DNA element carrying the iota toxin genes found in pCPPB-1.

In both iota toxin plasmid families, the site of the iota toxin gene insertion is the *cpe* promoter region. However, the site of this insertional event varied slightly in the two iota toxin plasmid families, with important consequences. In classical pCPF5603-like iota toxin plasmids, this insertional event silenced the *cpe* promoter, leading to loss of expression. In contrast, for the pCPPB-1 family, only one of three *cpe* promoters was lost during insertion of the iota toxin genes, so the *cpe* gene can still be transcribed. In pCPPB-1, two (P1 and P2) *cpe* promoters are still present upstream of the *cpe* ORF, while the third *cpe* promoter (P3) is missing (Figure S6). Since the P1 and P2 promoters are the major promoters, while the P3 promoter is less active, this explains why the *cpe* gene of pCPPB-1 is still transcribed [18], [19].

MLST analysis using chromosomal house-keeping genes revealed that the four novel type E isolates carrying pCPPB-1-like plasmids share a close relationship, but are not related to the classical type E strains carrying pCPF5603-related iota toxin plasmids or with type B, C, or D strain animal disease strains (Figure 5). These findings suggest that type E isolates carrying pCPPB-1-like plasmids represent a unique cluster of *C. perfringens* strains. In a recent survey, *C. perfringens* isolates from freshwater suspended sediment and sewage contained *cpe*-positive isolates, but many of those isolates had a different *cpe* locus from the previously characterized *cpe* loci [3], [5]. Some of these might be type E isolates with pCPPB-1-like plasmids. Another recent study identified putative *cpe*-positive type A isolates, which did not classify into known *cpe*-genotypes, that produce a cytotoxic factor(s) [6]. These strains could also resemble the type E strains identified in this study.

The findings in this study have clinical diagnostic significance. The multiplex PCR-based toxin genotyping assay is widely used to type *C. perfringens* clinical or veterinary disease isolates. Our findings indicate that the current multiplex PCR assay is not completely reliable, as it will misidentify as type A strains the newly discovered type E strains carrying pCPPB-1-like plasmids. The failure of the current multiplex PCR to detect these new pCPPB-1-carrying type E strains is due to sequence differences in the *iap* gene region used for primer design. Previously type E isolates were not identified in Japan, but the current study has now identified probable type E strains from Japan. Thus, type E strains may be more common than previously appreciated. Future studies should improve the reliability of the multiplex PCR by including primers capable of detecting the variant iota toxin genes identified in this study.

## Materials and Methods

### Bacterial strains and culture

Four *C. perfringen*s strains (PB-1, TGII002, TGII003, and 3441) used in the study were isolated from retail meat products in Japan, according to the methods previously described [7]. Several *C. perfringens* strains (F5603, F4969, NCTC8533, NCTC8081, NCTC8084 and ATCC13124) were used as positive or negative controls, as specified.

All strains were cultured in FTG (fluid thioglycolate; Difco Laboratories) medium, or TGY (3% tryptic soy broth [Difco], 2% glucose, 1% Yeast extract [Difco], and 0.1% L-Cysteine [Wako]) medium. For spore formation, Duncan-Strong medium was also used [7].

### Sequence of the *cpe* gene in isolates carrying an atypical *cpe* locus

A *cpe* genotyping PCR assay was first performed as described previously [5]. Since they contained unique *cpe* loci, the *cpe* sequences present in isolates PB-1, 3441, TGII002, and TGII003 were directly sequenced from PCR products amplified using primers (cpe-SD, cpe-DB, cpe-DB4, cpe-F3 and cpe-B3) that were derived from PB-1 sequence information (see Results and Table S1). Each PCR mixture contained 1 µl of template DNA preparation, 0.25 µl of GoTaq Flexi DNA polymerase (Promega), 2 µl of 2 mM NTPs, 4 µl of 25 mM MgCl_2_, 10 µl of PCR buffer, 2 µl of each primer pair (1 µM final concentration). The PCR reactions for determining *cpe* gene sequence were conducted in a iCycler (BioRad) using the following conditions: 94°C, for 2 min; 35 cycles of 94°C for 30 sec, 50°C for 60 sec, 68°C for 60 sec; and a single extension at 68°C for 8 min. DNA samples of all investigated strains were prepared with InstaGene Matrix kit (Bio-Rad) using a previously described method [7].

### Toxin genotyping and PCR survey of the representative plasmid-borne genes

The toxin genotype of investigated isolates was determined using a multiplex PCR genotyping assay, as previously reported [8].

A PCR assay of representative individual genes present on other known *C. perfringens* plasmids was also performed for isolates PB-1, TGII002, TGII003, and 3441. Using previously described primers [9], [10], [11], the genes investigated by PCR included the *rep* gene found on *cpe* plasmids pCPF4969 and pCPF5603, the *rep* gene carried on the *etx* plasmid pCP8533etx, and the *tcpF*, *cna*, *dcm*, *dam*, *soj*, *parB*, *capK*, *comEC*, *ThiF*, *spaC*, and *virS*-like genes found on pCPF4969 or pCPF5603. For investigating the presence of *tcp* genes (*tcpA* and *tcpH*), IS*1470*, IS*1151* and IS*1469*, newly constructed primers were used (Table S2). Each PCR mixture contained 1 µl of template DNA, 1 µl of *Tfi* DNA polymerase (Invitrogen), 2 µl of 2 mM NTPs, 1.5 µl of 50 mM MgCl_2_, 10 µl of PCR buffer, and 2 µl of each primer pair (1 µM final concentration). PCR reactions used the following conditions: 94°C, for 2 min; 35 cycles of 94°C for 30 sec, 55°C for 60 sec, 68°C for 60 sec; with a single extension at 68°C for 8 min.

### Pulsed-field gel electrophoresis

*C. perfringens* DNA plugs were prepared according to methods previously described [10]. Briefly, each strain was cultured in TGY medium for 16 to 18 hours at 37°C. Overnight TGY cultures were washed, pelleted, and then resuspended in TE buffer and mixed with 2% pulsed-field gel electrophoresis (PFGE)-certified agarose (Bio-Rad Laboratories), for a final agarose concentration of 1%. Those plugs were treated with acromoprotease (1 mg/ml [WAKO]) and then treated with proteinase K (1 µg/ml [Wako]). Treated plugs were electrophoresed in a CHEF-DR II PFGE system (Bio-Rad Laboratories) as previously described [10].

### Southern blot analysis of pulsed-field gels

Electrophoresed gels were exposed to UV in a CL-1000 ultraviolet crosslinker (UVP) to cut DNA and then denatured in sodium hydroxide solution (0.5 M NaOH, 1.5 M NaCl). DNA fragments in treated gels were then transferred to a positively charged Nylon membrane (Roche). Nylon membranes were cross-linked and then used for hybridization with DIG-labeled *cpe* probe as described previously using the PCR DIG probe synthesis kit (Roche) [12]. After hybridization with a probe, the pulsed-field Southern blots were developed using reagents from the DIG DNA detection kit (Roche).

### Sequencing of the PB-1 *cpe* plasmid

Using a previously described method [11], crude plasmid DNA was prepared from the PB-1 strain. To sequence the *cpe* plasmid in that strain, the crude plasmid DNA preparation was digested with restriction enzymes (XbaI or HindIII). Digested DNA fragments were then ligated into pBlueScript II SK^+^ vector and transformed into *E. coli* HST01 strain (TaKaRa) according to the methods described previously [11]. DNA inserts in randomly selected transformants were sequenced on both strands and the resultant sequences were subjected to BLAST analysis. When the acquired sequence was apparently derived from a plasmid, the inserted DNA fragment was completely sequenced and that DNA sequence information was used to construct contigs. The constructed contigs were then connected using long PCR assays. From these procedures, the *cpe* plasmid in PB-1 strain (pCPPB-1) was completely sequenced using sequencing primers (Table S3).

### PCR analysis of the locus containing toxin genes

To investigate the presence in other isolates of the iota toxin-like genes identified by sequencing the PB-1 *cpe* plasmid (see Results), a PCR assay was first performed using PB-1cpeB7 and PB-1 iAF primers for sequences in the variant *iap* gene and PB-1 iB-R2 and PB-1PCPF4 primers for sequences in the variant *ibp* gene (Table S1). This PCR mixture contained 1 µl of template DNA preparations, 1 µl of *Tfi Taq* polymerase (Invitrogen), 2 µl of 2 mM NTPs, 1.5 µl of 50 mM MgCl_2_, 10 µl of PCR buffer, and 2 µl of each primer pair (1 µM final concentration). PCR conditions were the same as used for PCR survey of plasmid genes.

To investigate the proximity of the variant *cpe*, *iap*, and *ibp* genes in isolates carrying a previously uncharacterized *cpe* locus, a PCR assay was performed with cpe-d2 and NISR1.3 primers for the *cpe* downstream region, cpe-B3 and PB-1 iaF primers for *cpe* and *iap*-like connection, and PB-1cpeB7 and PB-1 iB-R2 primers for *iap*-like and *ibp*-like connection (Table S1). Each PCR mixture contained 1 µl of template DNA preparation, 25 µl of PrimeStar Max polymerase Premix (TaKaRa), and 2 µl of each primer pair (1 µM final concentration). PCR reactions were performed under the following conditions: 94°C, for 2 min; 40 cycles of 94°C for 30 sec, 65°C (61°C for *cpe* and *cpe* downstream region assay) for 30 sec, 68°C for 120 sec; with a single extension at 68°C for 8 min.

Overlapping PCR assays for the locus carrying the variant *cpe* gene of PB-1 were performed. Primers used for investigating toxin loci are listed in Table S4. Each PCR mixture contained 1 µl of template DNA preparation, 0.2 µl of Platinum *Taq* polymerase (Invitrogen), 2 µl of 2 mM NTPs, 1.5 µl of 50 mM MgCl_2_, 5 µl of PCR buffer, and 2 µl of each primer pair (1 µM final concentration). PCR conditions used were: 1 cycle at 94°C for 2 min; 35 cycles at 94°C for 30 s, 58°C for 1 min, and 72°C for 100 s; and a single extension of 72°C for 10 min.

### PCR analyses to evaluate carriage of the pCPF4969 variable region and the transfer region

The PCR survey results and complete sequence analysis of the pCPPB-1 *cpe* plasmid indicated that the *cpe* locus is harbored on a plasmid in three other food isolates and suggested those plasmids might possess a similar variable region as pCPPB-1 and pCPF4969, which is a classical *cpe* plasmid. To evaluate the similarities of variable regions of the *cpe* plasmids in these three isolates, overlapping PCR analyses were performed with the same primers that had been used for evaluating the *cpe* plasmid in soil isolates carrying an atypical *cpe* locus [2], [11]. Also, to evaluate the presence of a putative plasmid transfer region in these three isolates, overlapping PCR assays were performed using previously described primers and newly constructed primers based on pCPPB-1 sequence data obtained in this study (Table S5) [2], [11], [13].

Each PCR mixture contained 1 µl of template DNA preparation, 0.2 µl of Platinum *Taq* polymerase (Invitrogen), 2 µl of 2 mM NTPs, 1.5 µl of 50 mM MgCl_2_, 5 µl of PCR buffer, and 2 µl of each primer pair (1 µM final concentration). PCR conditions used included: 1 cycle at 94°C for 2 min; 35 cycles at 94°C for 30 s, 58°C for 1 min, and 72°C for 100 s; with a single extension of 72°C for 10 min.

### Total RNA preparations and RT-PCR analysis of toxin genes

From results of sequencing analyses and PCR assays for toxin genes, PB-1, TGII002, TGII003, and 3441 were each found to carry a new toxin locus containing variant *cpe* and iota toxin genes (see Results). The expression of these variant toxin genes were investigated with RT-PCR assays using cpe-F3 and cpe-B3 primers for the *cpe* gene, PB-1cpeB7 and PB-1 iA-F primers for the variant *iap* gene, and PB-1 iB-R2 and PB-1PCPF4 primers for the variant *ibp*- gene (Table S1). For total RNA preparations, 10 ml culture specimens of TGY media (4 hours) or DS media (18 to 24 hours) were collected by centrifugation at 5,000 g for 10 min. After the supernatant was discarded, 5 ml of RNAlater reagent (Ambion) were added and mixed with a vortex mixture, and then stored at 4°C. Total RNA was extracted from stored bacteria using the RNeasy Mini kit (QIAGEN) according to the manufacturer's instructions. Prepared RNA samples were then treated with TURBO DNA-free (Ambion) to remove contaminated DNA according to the manufacturer's instructions.

RNA preparations (1 µl of aliquot) obtained from TGY cultures were used for RT-PCR assays detecting the *iap* and *ibp* toxin mRNA, and RNA preparations (4 µl of aliquot) from Duncan-Strong media were used for detecting the *cpe* mRNA and the *sigK* mRNA (for confirming spore formation). RT-PCR assays were performed using Superscript III One-Step RT-PCR with Platinum *Taq* (Invitrogen). Reaction mixture contained DNA template, 25 µl of Reaction Mix, 2 µl of each primer pair (1 µM final concentration), and 2 µl of SuperScript III RT/Platinum *Taq* Mix, The reaction program for reverse transcription (one cycle at 45°C for 30 min) used the following PCR amplification program: 1st cycle 94°C for 5 min; 2nd to 35th cycles, 94°C for 30 s, 55°C for 30 s, and 68°C for 1 min; 36th cycle, and 68°C for 8 min. For detecting the possible presence of contaminating DNA, a PCR reaction was performed with Platinum *Taq* DNA polymerase and the same PCR amplification program as described above. Reaction mixture contained template RNA preparations, 0.2 µl of Platinum *Taq* DNA polymerase (Invitrogen), 2 µl of 2 mM NTPs, 1.5 µl of 50 mM MgCl_2_, 10 µl of PCR buffer, and 2 µl of each primer pair (1 µM final concentration).

### Sequence analysis of the *ssp4* gene and assessment of spore heat resistance properties

To help evaluate potential heat resistance properties of spores made by type E isolates carrying the variant *cpe* gene, sequencing analysis was first performed, as described [14], [15], for the *ssp4* gene, which is a very important gene affecting heat resistance of *C. perfringens* spores. The D value at 100°C for spores of strains PB-1 and 3441 were also determined according to methods previously described [7]. The D value of spores made by other isolates at 100°C was not determined, because spore formation by TGII002, and TGII003 strains was highly variable from experiment to experiment.

### Nucleotide sequence accession numbers

The complete pCPPB-1 sequence was deposited in GenBank under accession number AB604032. The sequence of the variant *cpe* genes present in the four surveyed isolates were deposited under accession number AB604033 to AB604035, and the sequence of the *ssp4* gene in the four isolates were deposited under accession number AB604038 to AB604041. The sequences from MLST analysis can be located under accession number AB604044 to to AB604091.

## Supporting Information

Figure S1 **Alignment of deduced amino acid substitutions in the variant** ***cpe*** **ORF.** Putative CPE amino acid substitutions encoded by strains PB-1 were highly homologous with the previously known *cpe* gene, especially in the receptor binding region and the major cytotoxicity region. Different amino acid substitutions are indicated as a bold letter.
(PPT)

Figure S2 **Analysis of variant Ia component of iota toxin that is putatively produced by strain PB-1.** Upper portion shows putative functional regions. Lower portion shows comparison of deduced amino acid sequence of the iota toxin Ia component encoded by pCPPB-1, *C. perfringens* type E strain (JGS1987), or the activity component of CdtA toxin in *C. difficile*.
(PPT)

Figure S3 **Analysis of the Ib component of iota toxin that is putatively produced by the PB-1 strain.** Upper portion shows putative functional regions. Lower portion shows comparison of deduced amino acid sequence among iota toxin Ib component on pCPPB-1, Ib component in *C. perfringens* type E strain (JGS1987), and the CdtB binding component of CDT in *C. difficile*.
(PPT)

Figure S4 **Comparison of nucleotide sequence of the variant** ***iap*** **gene of pCPPB-1 against the** ***iap*** **gene of type E isolate (JGS1987).** Blue bar indicates primer sites using multiplex PCR toxin genotyping assay [8].
(PPT)

Figure S5 **Comparison of nucleotide sequence of the variant** ***ibp*** **gene of pCPPB-1 against the** ***ibp*** **gene of classical type E isolates (JGS1987).**
(PPT)

Figure S6 **Schematic presentation showing putative** ***cpe*** **promoter sequences in the chromosomal** ***cpe*** **strain NCTC10240 versus those in the the PB-1 strain carrying the plasmid borne variant** ***cpe*** **gene.**
(PPT)

Figure S7 **Overlapping PCR assays of plasmid encoding the variant** ***cpe*** **gene in four isolates using primers designed to amplify the pCPF5603/pCPPB-1 transfer region.** Shown are results obtained using DNA from strains (PB-1, 3441, TGII002 and TGII003), which carry the plasmid-borne variant *cpe* gene or from F4969 and F5603 which carry the classical *cpe* plasmids pCPF4969 and pCPF5603. Using primers previously described, the region assayed with PCR reaction (T6 to T16) contained the eight *tcp* (*tcpA* to *tcpI*) genes and the *intP* gene, which genes are thought to be necessary for plasmid transfer (Table S4) [11], [13]. The overlapping PCR (reaction T14t to T16t) used newly constructed primers based on the sequence information of the *tcpA* region on pCPPB-1 (Table S4).
(PPT)

Figure S8 **Overlapping PCR assays of plasmid carrying the variant** ***cpe*** **gene in four isolates using primers designed to amplify the pCPF4969 variable region.** Shown are results using DNA specimens from strains (PB-1, 3441, TGII002 and TGII003), which carries the variant *cpe* gene or from F4969 and F5603, which are strains carrying the classical *cpe* plasmid pCPF4969 and pCPF5603 [11].
(PPT)

Figure S9 **Overlapping PCR assays of the plasmids encoding the variant** ***cpe*** **gene in four isolates using primers designed to amplify the toxin region.** Shown are results using DNA from strains (PB-1, 3441, TGII002 and TGII003), which carries the variant *cpe* gene and variant iota genes, and from NCTC8084 type E strain carrying the silent *cpe* gene and iota genes.
(PPT)

Table S1 **Primers for toxin region assays.**
(XLS)

Table S2 **Primers for PCR survey of 18 genes.**
(XLS)

Table S3 **Primers for plasmid sequencing.**
(XLS)

Table S4 **Primers for overlapping PCR assays for pCPPB-1 iota-like toxin region PCR.**
(XLS)

Table S5 **Primers for pCPPB-1 transfer region PCR.**
(XLS)
