# Supplementary material for: Identification of Novel Clostridium perfringens Type E Strains That Carry an Iota Toxin Plasmid with a Functional Enterotoxin Gene
Source: PLoS One. 2011 May 31;6(5):e20376. doi: 10.1371/journal.pone.0020376 (PMC3105049; doi:10.1371/journal.pone.0020376)
Supplement: Figure S1 — Alignment of deduced amino acid substitutions in the variant cpe ORF. Putative CPE amino acid substitutions encoded by strains PB-1 were highly homologous with the previously known cpe gene, especially in the receptor binding region and the major cytotoxicity region. Different amino acid substitutions are indicated as a bold letter. (PPT) [file pone.0020376.s001.ppt]

## Slide 1
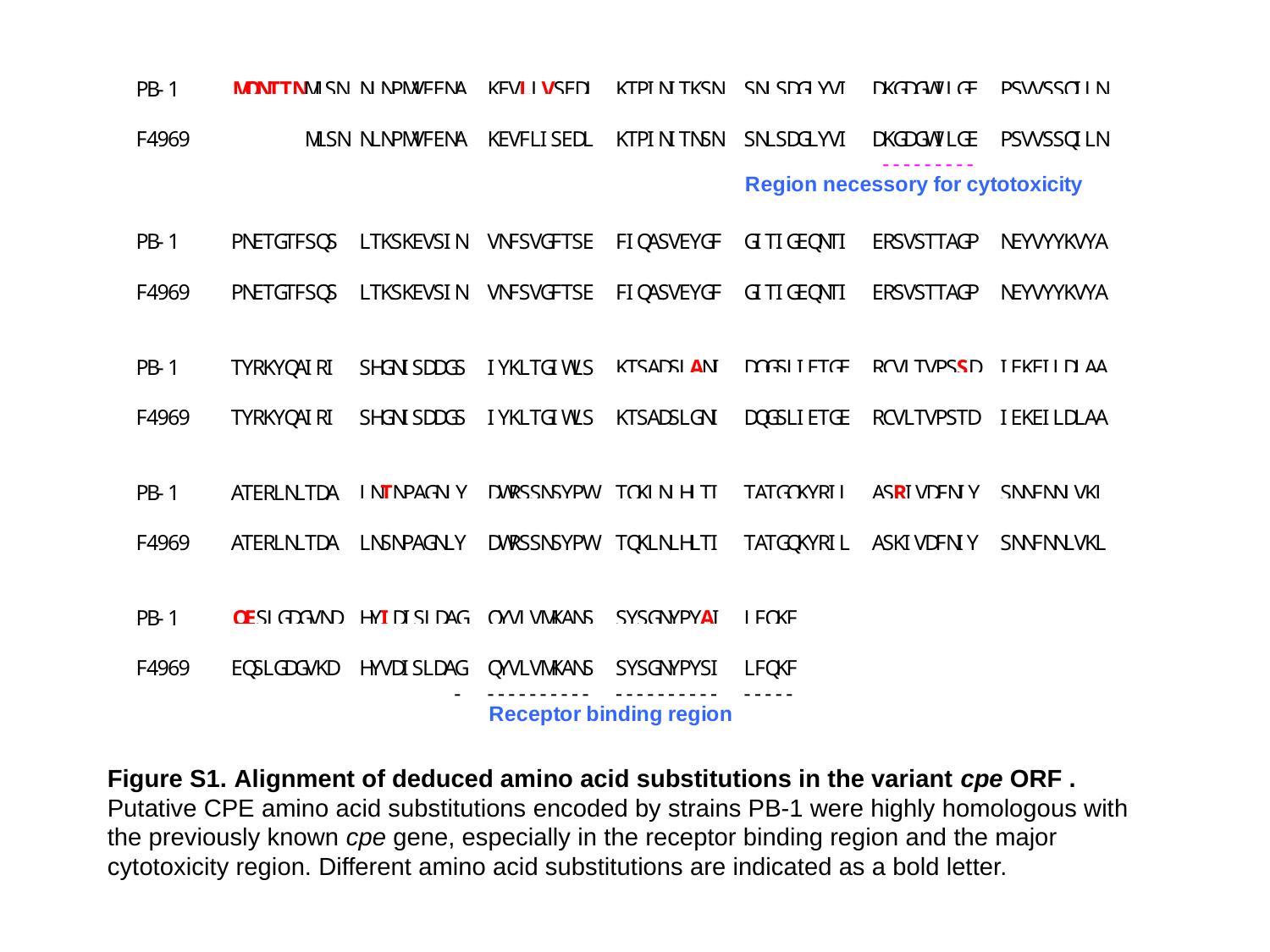

Figure S1. Alignment of deduced amino acid substitutions in the variant cpe ORF . Putative CPE amino acid substitutions encoded by strains PB-1 were highly homologous with the previously known cpe gene, especially in the receptor binding region and the major cytotoxicity region. Different amino acid substitutions are indicated as a bold letter.
