# Supplementary material for: Identification of Novel Clostridium perfringens Type E Strains That Carry an Iota Toxin Plasmid with a Functional Enterotoxin Gene
Source: PLoS One. 2011 May 31;6(5):e20376. doi: 10.1371/journal.pone.0020376 (PMC3105049; doi:10.1371/journal.pone.0020376)
Supplement: Figure S2 — Analysis of variant Ia component of iota toxin that is putatively produced by strain PB-1. Upper portion shows putative functional regions. Lower portion shows comparison of deduced amino acid sequence of the iota toxin Ia component encoded by pCPPB-1, C. perfringens type E strain (JGS1987), or the activity component of CdtA toxin in C. difficile. (PPT) [file pone.0020376.s002.ppt]

## Slide 1
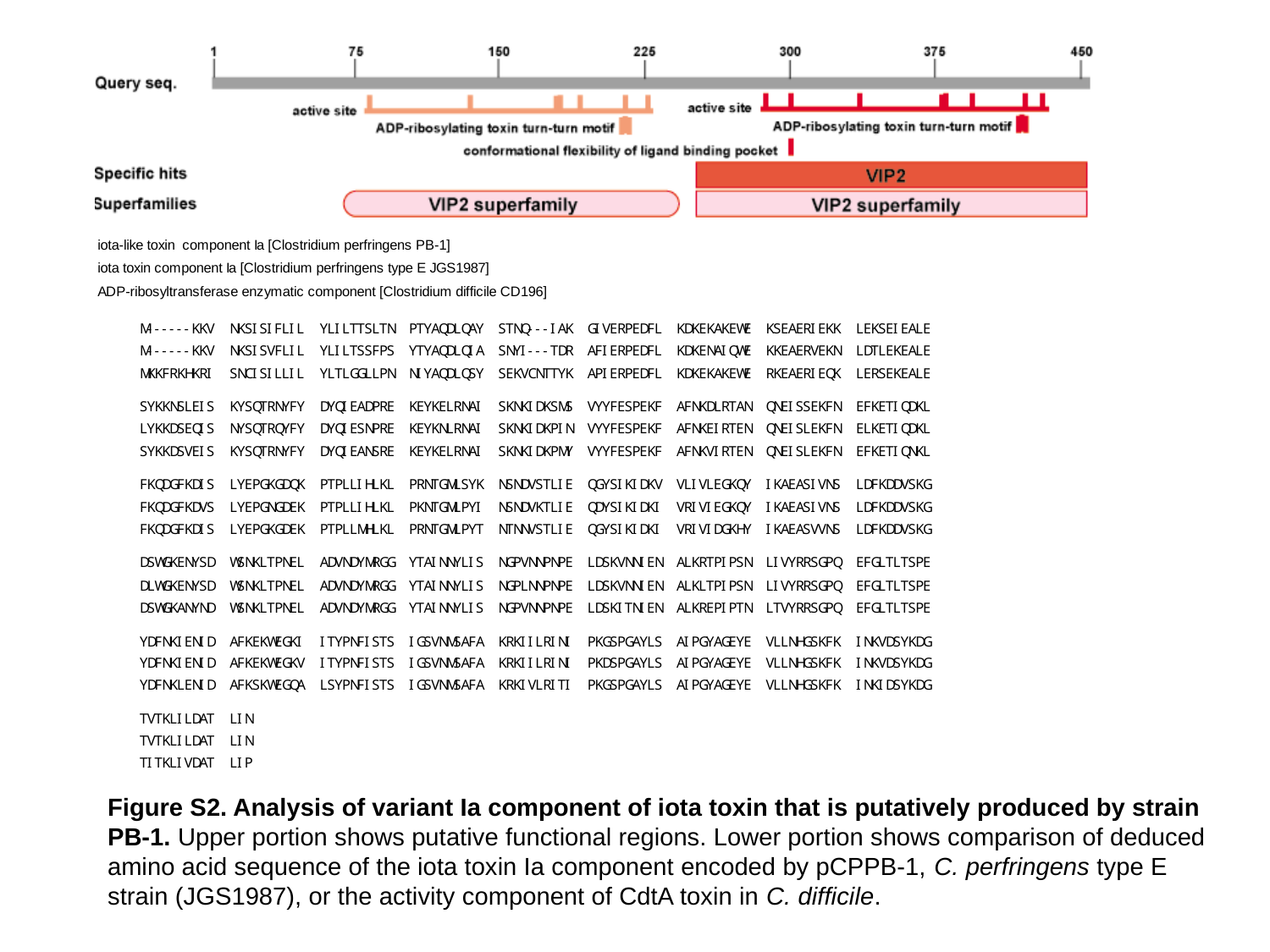

Figure S2. Analysis of variant Ia component of iota toxin that is putatively produced by strain PB-1. Upper portion shows putative functional regions. Lower portion shows comparison of deduced amino acid sequence of the iota toxin Ia component encoded by pCPPB-1, C. perfringens type E strain (JGS1987), or the activity component of CdtA toxin in C. difficile.
