# Supplementary material for: Identification of Novel Clostridium perfringens Type E Strains That Carry an Iota Toxin Plasmid with a Functional Enterotoxin Gene
Source: PLoS One. 2011 May 31;6(5):e20376. doi: 10.1371/journal.pone.0020376 (PMC3105049; doi:10.1371/journal.pone.0020376)
Supplement: Figure S3 — Analysis of the Ib component of iota toxin that is putatively produced by the PB-1 strain. Upper portion shows putative functional regions. Lower portion shows comparison of deduced amino acid sequence among iota toxin Ib component on pCPPB-1, Ib component in C. perfringens type E strain (JGS1987), and the CdtB binding component of CDT in C. difficile. (PPT) [file pone.0020376.s003.ppt]

## Slide 1
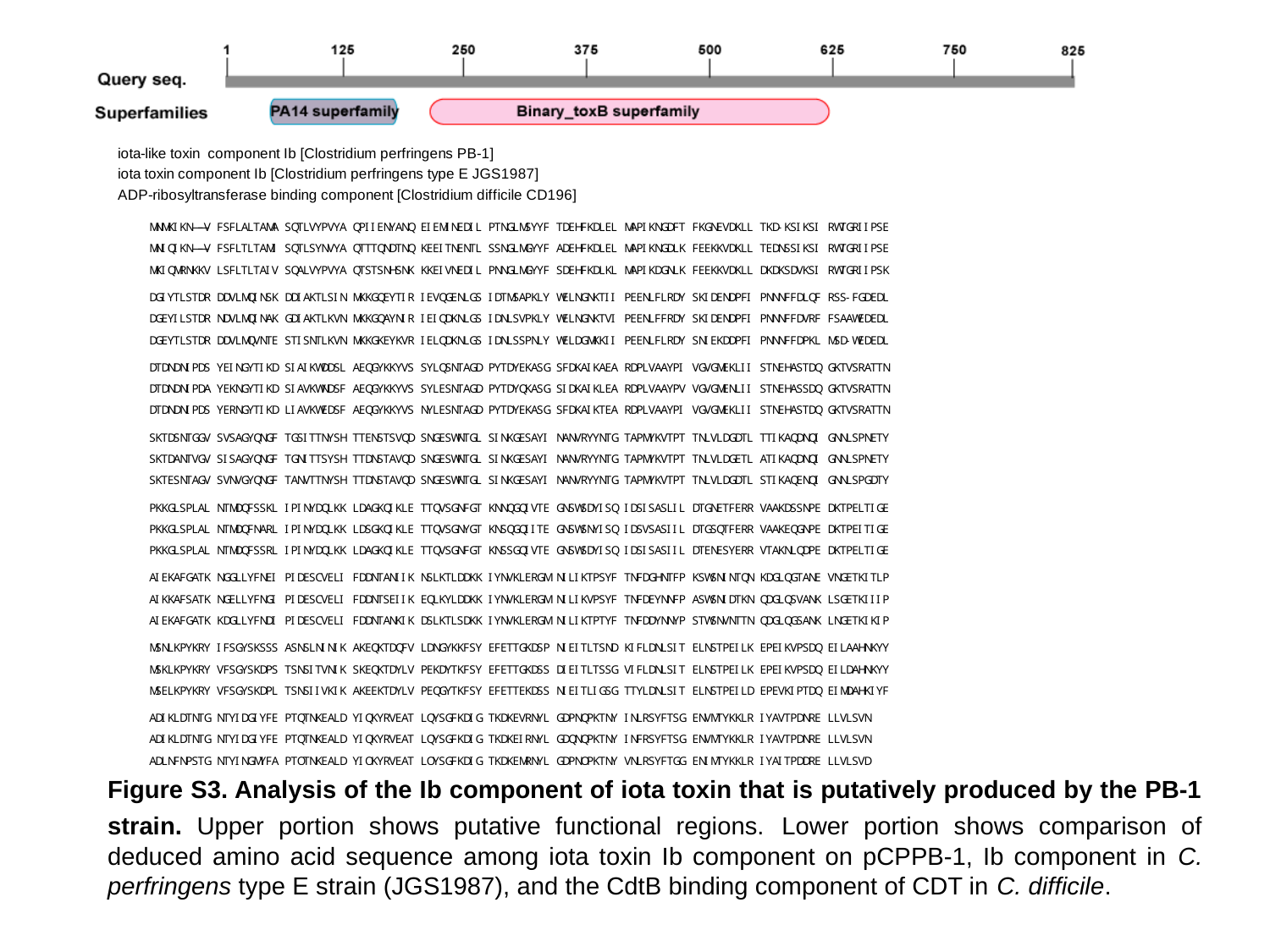

Figure S3. Analysis of the Ib component of iota toxin that is putatively produced by the PB-1 strain. Upper portion shows putative functional regions. Lower portion shows comparison of deduced amino acid sequence among iota toxin Ib component on pCPPB-1, Ib component in C. perfringens type E strain (JGS1987), and the CdtB binding component of CDT in C. difficile.
