# Supplementary material for: Identification of Novel Clostridium perfringens Type E Strains That Carry an Iota Toxin Plasmid with a Functional Enterotoxin Gene
Source: PLoS One. 2011 May 31;6(5):e20376. doi: 10.1371/journal.pone.0020376 (PMC3105049; doi:10.1371/journal.pone.0020376)
Supplement: Figure S4 — Comparison of nucleotide sequence of the variant iap gene of pCPPB-1 against the iap gene of type E isolate (JGS1987). Blue bar indicates primer sites using multiplex PCR toxin genotyping assay [8]. (PPT) [file pone.0020376.s004.ppt]

## Slide 1
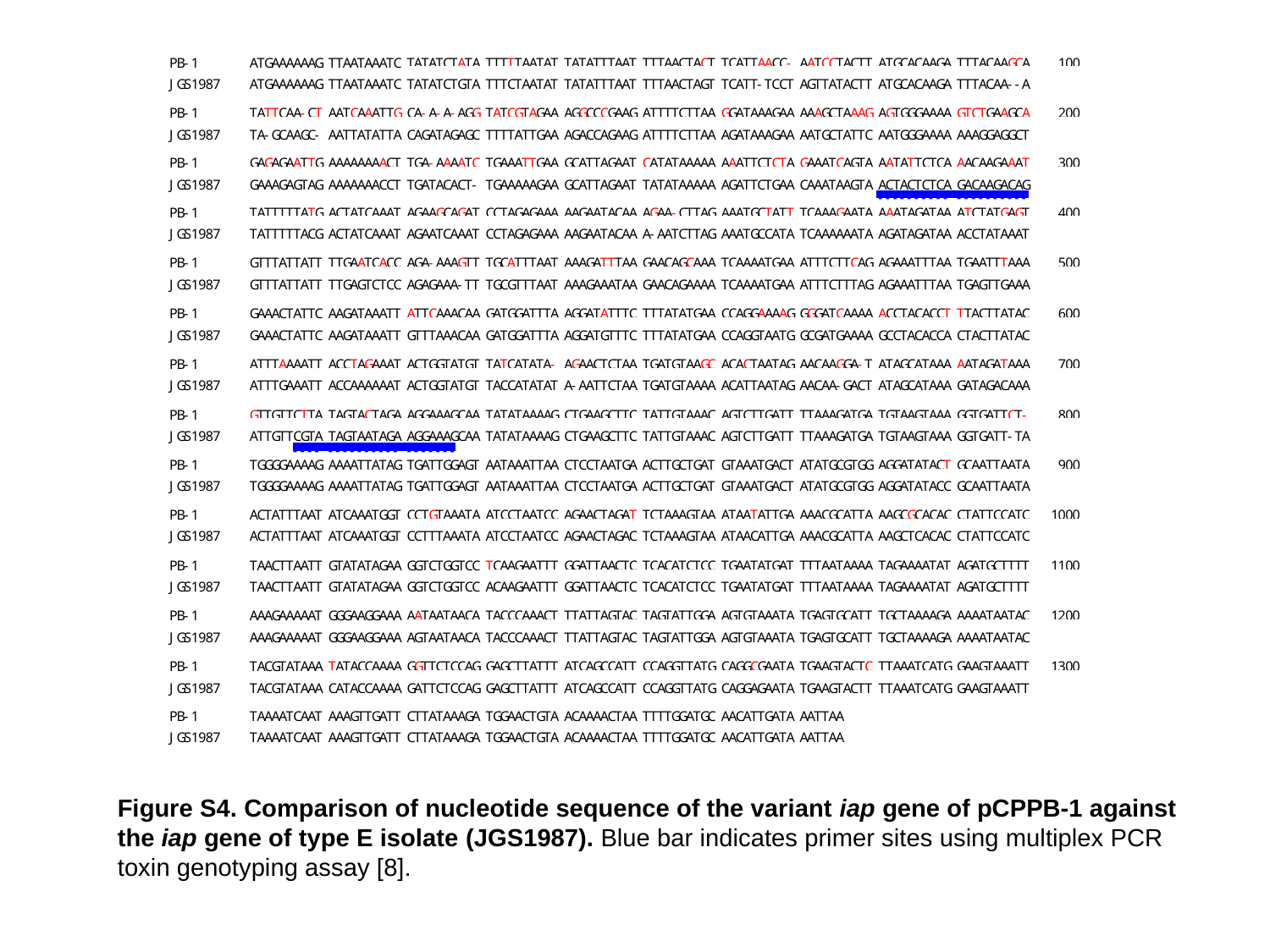

Figure S4. Comparison of nucleotide sequence of the variant iap gene of pCPPB-1 against the iap gene of type E isolate (JGS1987). Blue bar indicates primer sites using multiplex PCR toxin genotyping assay [8].
