# Supplementary material for: Identification of Novel Clostridium perfringens Type E Strains That Carry an Iota Toxin Plasmid with a Functional Enterotoxin Gene
Source: PLoS One. 2011 May 31;6(5):e20376. doi: 10.1371/journal.pone.0020376 (PMC3105049; doi:10.1371/journal.pone.0020376)
Supplement: Figure S5 — Comparison of nucleotide sequence of the variant ibp gene of pCPPB-1 against the ibp gene of classical type E isolates (JGS1987). (PPT) [file pone.0020376.s005.ppt]

## Slide 1
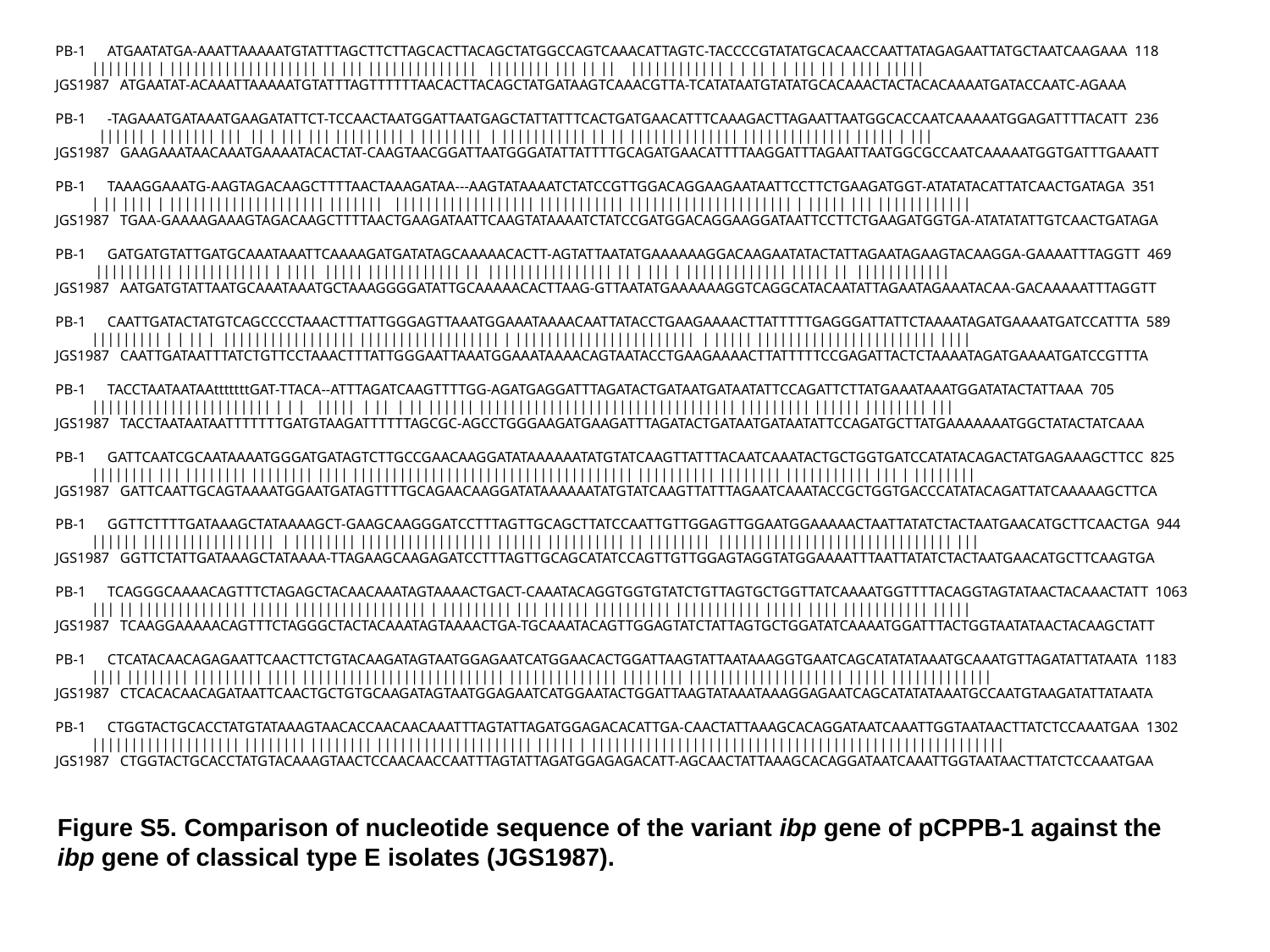

PB-1 ATGAATATGA-AAATTAAAAATGTATTTAGCTTCTTAGCACTTACAGCTATGGCCAGTCAAACATTAGTC-TACCCCGTATATGCACAACCAATTATAGAGAATTATGCTAATCAAGAAA 118
 |||||||| | ||||||||||||||||||| || ||| |||||||||||||| |||||||| ||| || || |||||||||||| | | || | | ||| || | |||| |||||
JGS1987 ATGAATAT-ACAAATTAAAAATGTATTTAGTTTTTTAACACTTACAGCTATGATAAGTCAAACGTTA-TCATATAATGTATATGCACAAACTACTACACAAAATGATACCAATC-AGAAA
PB-1 -TAGAAATGATAAATGAAGATATTCT-TCCAACTAATGGATTAATGAGCTATTATTTCACTGATGAACATTTCAAAGACTTAGAATTAATGGCACCAATCAAAAATGGAGATTTTACATT 236
 |||||| | ||||||| ||| || | ||| ||| ||||||||| | |||||||| | ||||||||||| || || |||||||||||||| |||||||||||||| ||||| | |||
JGS1987 GAAGAAATAACAAATGAAAATACACTAT-CAAGTAACGGATTAATGGGATATTATTTTGCAGATGAACATTTTAAGGATTTAGAATTAATGGCGCCAATCAAAAATGGTGATTTGAAATT
PB-1 TAAAGGAAATG-AAGTAGACAAGCTTTTAACTAAAGATAA---AAGTATAAAATCTATCCGTTGGACAGGAAGAATAATTCCTTCTGAAGATGGT-ATATATACATTATCAACTGATAGA 351
 | || |||| | |||||||||||||||||||| ||||||| |||||||||||||||||| ||||||||||| ||||||||||||||||||||| | ||||| ||| ||||||||||||
JGS1987 TGAA-GAAAAGAAAGTAGACAAGCTTTTAACTGAAGATAATTCAAGTATAAAATCTATCCGATGGACAGGAAGGATAATTCCTTCTGAAGATGGTGA-ATATATATTGTCAACTGATAGA
PB-1 GATGATGTATTGATGCAAATAAATTCAAAAGATGATATAGCAAAAACACTT-AGTATTAATATGAAAAAAGGACAAGAATATACTATTAGAATAGAAGTACAAGGA-GAAAATTTAGGTT 469
 |||||||||| |||||||||||| | |||| ||||| |||||||||||| || |||||||||||||||| || | ||| | ||||||||||||| ||||| || ||||||||||||
JGS1987 AATGATGTATTAATGCAAATAAATGCTAAAGGGGATATTGCAAAAACACTTAAG-GTTAATATGAAAAAAGGTCAGGCATACAATATTAGAATAGAAATACAA-GACAAAAATTTAGGTT
PB-1 CAATTGATACTATGTCAGCCCCTAAACTTTATTGGGAGTTAAATGGAAATAAAACAATTATACCTGAAGAAAACTTATTTTTGAGGGATTATTCTAAAATAGATGAAAATGATCCATTTA 589
 ||||||||| | | || | ||||||||||||||||| |||||||||||||||||| | ||||||||||||||||||||||| | ||||| ||||||||||||||||||||||| ||||
JGS1987 CAATTGATAATTTATCTGTTCCTAAACTTTATTGGGAATTAAATGGAAATAAAACAGTAATACCTGAAGAAAACTTATTTTTCCGAGATTACTCTAAAATAGATGAAAATGATCCGTTTA
PB-1 TACCTAATAATAAtttttttGAT-TTACA--ATTTAGATCAAGTTTTGG-AGATGAGGATTTAGATACTGATAATGATAATATTCCAGATTCTTATGAAATAAATGGATATACTATTAAA 705
 ||||||||||||||||||||||| | | | ||||| | || | || |||||| ||||||||||||||||||||||||||||||||| ||||||||| |||||| |||||||| |||
JGS1987 TACCTAATAATAATTTTTTTGATGTAAGATTTTTTAGCGC-AGCCTGGGAAGATGAAGATTTAGATACTGATAATGATAATATTCCAGATGCTTATGAAAAAAATGGCTATACTATCAAA
PB-1 GATTCAATCGCAATAAAATGGGATGATAGTCTTGCCGAACAAGGATATAAAAAATATGTATCAAGTTATTTACAATCAAATACTGCTGGTGATCCATATACAGACTATGAGAAAGCTTCC 825
 |||||||| ||| |||||||| |||||||| |||| |||||||||||||||||||||||||||||||||||| |||||||||| |||||||| ||||||||||| ||| | ||||||||
JGS1987 GATTCAATTGCAGTAAAATGGAATGATAGTTTTGCAGAACAAGGATATAAAAAATATGTATCAAGTTATTTAGAATCAAATACCGCTGGTGACCCATATACAGATTATCAAAAAGCTTCA
PB-1 GGTTCTTTTGATAAAGCTATAAAAGCT-GAAGCAAGGGATCCTTTAGTTGCAGCTTATCCAATTGTTGGAGTTGGAATGGAAAAACTAATTATATCTACTAATGAACATGCTTCAACTGA 944
 |||||| ||||||||||||||||| | |||||||| ||||||||||||||||| |||||| |||||||||| || |||||||| |||||||||||||||||||||||||||||| |||
JGS1987 GGTTCTATTGATAAAGCTATAAAA-TTAGAAGCAAGAGATCCTTTAGTTGCAGCATATCCAGTTGTTGGAGTAGGTATGGAAAATTTAATTATATCTACTAATGAACATGCTTCAAGTGA
PB-1 TCAGGGCAAAACAGTTTCTAGAGCTACAACAAATAGTAAAACTGACT-CAAATACAGGTGGTGTATCTGTTAGTGCTGGTTATCAAAATGGTTTTACAGGTAGTATAACTACAAACTATT 1063
 ||| || |||||||||||||| ||||| ||||||||||||||||| | ||||||||| ||| |||||| |||||||||| ||||||||||| ||||| |||| ||||||||||| |||||
JGS1987 TCAAGGAAAAACAGTTTCTAGGGCTACTACAAATAGTAAAACTGA-TGCAAATACAGTTGGAGTATCTATTAGTGCTGGATATCAAAATGGATTTACTGGTAATATAACTACAAGCTATT
PB-1 CTCATACAACAGAGAATTCAACTTCTGTACAAGATAGTAATGGAGAATCATGGAACACTGGATTAAGTATTAATAAAGGTGAATCAGCATATATAAATGCAAATGTTAGATATTATAATA 1183
 |||| |||||||| ||||||||| |||| |||||||||||||||||||||||||| |||||||||||||| |||||||| |||||||||||||||||||| ||||| |||||||||||||
JGS1987 CTCACACAACAGATAATTCAACTGCTGTGCAAGATAGTAATGGAGAATCATGGAATACTGGATTAAGTATAAATAAAGGAGAATCAGCATATATAAATGCCAATGTAAGATATTATAATA
PB-1 CTGGTACTGCACCTATGTATAAAGTAACACCAACAACAAATTTAGTATTAGATGGAGACACATTGA-CAACTATTAAAGCACAGGATAATCAAATTGGTAATAACTTATCTCCAAATGAA 1302
 ||||||||||||||||||| |||||||| |||||||| |||||||||||||||||||| ||||| | |||||||||||||||||||||||||||||||||||||||||||||||||||||
JGS1987 CTGGTACTGCACCTATGTACAAAGTAACTCCAACAACCAATTTAGTATTAGATGGAGAGACATT-AGCAACTATTAAAGCACAGGATAATCAAATTGGTAATAACTTATCTCCAAATGAA
Figure S5. Comparison of nucleotide sequence of the variant ibp gene of pCPPB-1 against the ibp gene of classical type E isolates (JGS1987).

## Slide 2
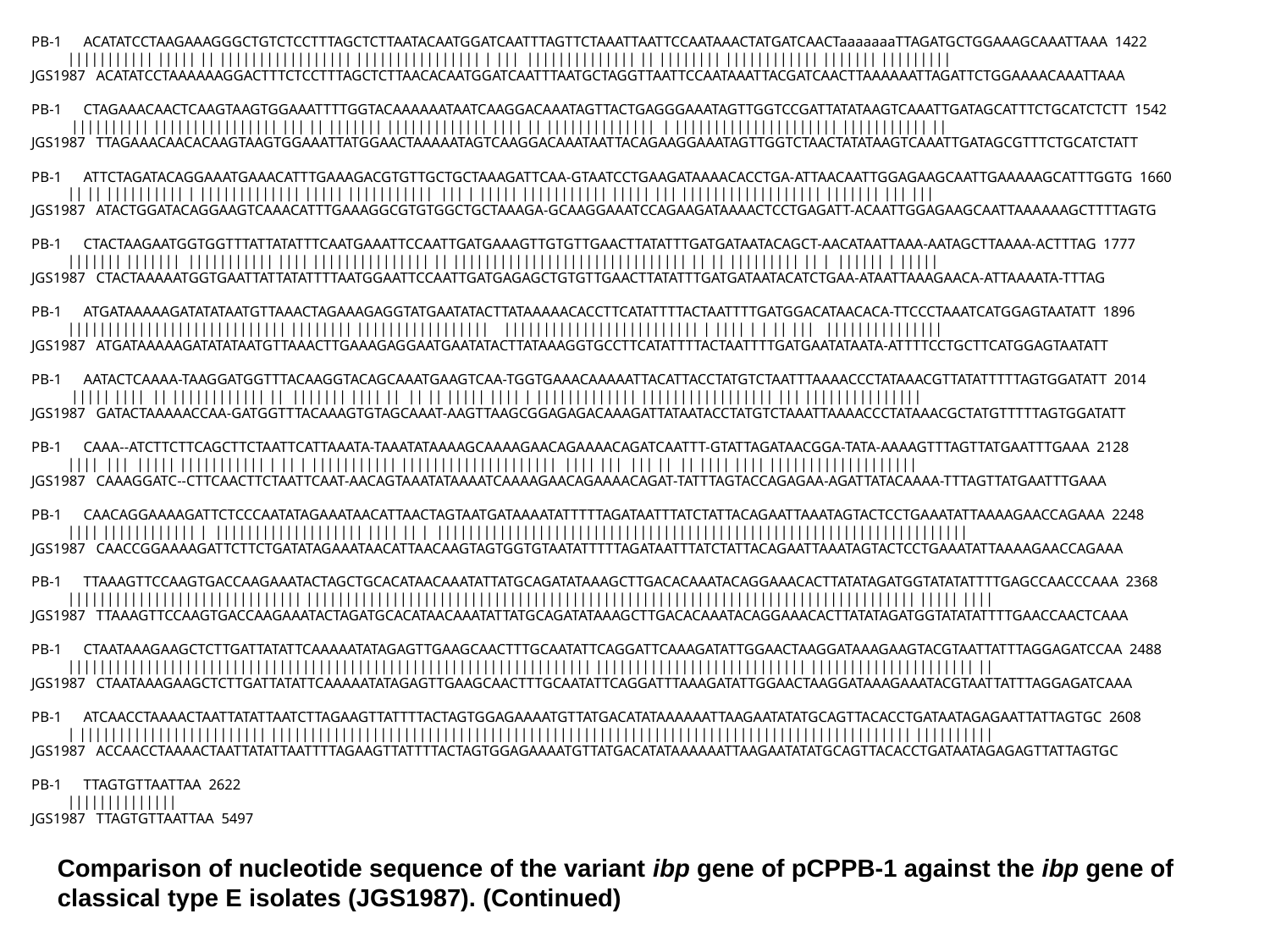

PB-1 ACATATCCTAAGAAAGGGCTGTCTCCTTTAGCTCTTAATACAATGGATCAATTTAGTTCTAAATTAATTCCAATAAACTATGATCAACTaaaaaaaTTAGATGCTGGAAAGCAAATTAAA 1422
 ||||||||||| ||||| || ||||||||||||||||| |||||||||||||||| | ||| |||||||||||||| || |||||||| |||||||||||| ||||||| |||||||||
JGS1987 ACATATCCTAAAAAAGGACTTTCTCCTTTAGCTCTTAACACAATGGATCAATTTAATGCTAGGTTAATTCCAATAAATTACGATCAACTTAAAAAATTAGATTCTGGAAAACAAATTAAA
PB-1 CTAGAAACAACTCAAGTAAGTGGAAATTTTGGTACAAAAAATAATCAAGGACAAATAGTTACTGAGGGAAATAGTTGGTCCGATTATATAAGTCAAATTGATAGCATTTCTGCATCTCTT 1542
 |||||||||| |||||||||||||||| ||| || ||||||| ||||||||||||| |||| || |||||||||||||| | ||||||||||||||||||||| ||||||||||| ||
JGS1987 TTAGAAACAACACAAGTAAGTGGAAATTATGGAACTAAAAATAGTCAAGGACAAATAATTACAGAAGGAAATAGTTGGTCTAACTATATAAGTCAAATTGATAGCGTTTCTGCATCTATT
PB-1 ATTCTAGATACAGGAAATGAAACATTTGAAAGACGTGTTGCTGCTAAAGATTCAA-GTAATCCTGAAGATAAAACACCTGA-ATTAACAATTGGAGAAGCAATTGAAAAAGCATTTGGTG 1660
 || || |||||||||| | ||||||||||||| ||||| ||||||||||| ||| | ||||| ||||||||||| ||||| ||| |||||||||||||||||| ||||||| ||| |||
JGS1987 ATACTGGATACAGGAAGTCAAACATTTGAAAGGCGTGTGGCTGCTAAAGA-GCAAGGAAATCCAGAAGATAAAACTCCTGAGATT-ACAATTGGAGAAGCAATTAAAAAAGCTTTTAGTG
PB-1 CTACTAAGAATGGTGGTTTATTATATTTCAATGAAATTCCAATTGATGAAAGTTGTGTTGAACTTATATTTGATGATAATACAGCT-AACATAATTAAA-AATAGCTTAAAA-ACTTTAG 1777
 ||||||| ||||||| ||||||||||| |||| ||||||||||||||| || |||||||||||||||||||||||||||||| || || ||||||||| || | |||||| | |||||
JGS1987 CTACTAAAAATGGTGAATTATTATATTTTAATGGAATTCCAATTGATGAGAGCTGTGTTGAACTTATATTTGATGATAATACATCTGAA-ATAATTAAAGAACA-ATTAAAATA-TTTAG
PB-1 ATGATAAAAAGATATATAATGTTAAACTAGAAAGAGGTATGAATATACTTATAAAAACACCTTCATATTTTACTAATTTTGATGGACATAACACA-TTCCCTAAATCATGGAGTAATATT 1896
 |||||||||||||||||||||||||||| |||||||| ||||||||||||||||| ||||||||||||||||||||||||| | |||| | | || ||| |||||||||||||||
JGS1987 ATGATAAAAAGATATATAATGTTAAACTTGAAAGAGGAATGAATATACTTATAAAGGTGCCTTCATATTTTACTAATTTTGATGAATATAATA-ATTTTCCTGCTTCATGGAGTAATATT
PB-1 AATACTCAAAA-TAAGGATGGTTTACAAGGTACAGCAAATGAAGTCAA-TGGTGAAACAAAAATTACATTACCTATGTCTAATTTAAAACCCTATAAACGTTATATTTTTAGTGGATATT 2014
 ||||| |||| || |||||||||||| || ||||||| |||| || || || ||||| |||| | ||||||||||||| ||||||||||||||||| ||| |||||||||||||||
JGS1987 GATACTAAAAACCAA-GATGGTTTACAAAGTGTAGCAAAT-AAGTTAAGCGGAGAGACAAAGATTATAATACCTATGTCTAAATTAAAACCCTATAAACGCTATGTTTTTAGTGGATATT
PB-1 CAAA--ATCTTCTTCAGCTTCTAATTCATTAAATA-TAAATATAAAAGCAAAAGAACAGAAAACAGATCAATTT-GTATTAGATAACGGA-TATA-AAAAGTTTAGTTATGAATTTGAAA 2128
 |||| ||| ||||| ||||||||||| | || | ||||||||||| |||||||||||||||||||| |||| ||| ||| || || |||| |||| |||||||||||||||||||
JGS1987 CAAAGGATC--CTTCAACTTCTAATTCAAT-AACAGTAAATATAAAATCAAAAGAACAGAAAACAGAT-TATTTAGTACCAGAGAA-AGATTATACAAAA-TTTAGTTATGAATTTGAAA
PB-1 CAACAGGAAAAGATTCTCCCAATATAGAAATAACATTAACTAGTAATGATAAAATATTTTTAGATAATTTATCTATTACAGAATTAAATAGTACTCCTGAAATATTAAAAGAACCAGAAA 2248
 |||| |||||||||||| | ||||||||||||||||||| |||| || | ||||||||||||||||||||||||||||||||||||||||||||||||||||||||||||||||||||
JGS1987 CAACCGGAAAAGATTCTTCTGATATAGAAATAACATTAACAAGTAGTGGTGTAATATTTTTAGATAATTTATCTATTACAGAATTAAATAGTACTCCTGAAATATTAAAAGAACCAGAAA
PB-1 TTAAAGTTCCAAGTGACCAAGAAATACTAGCTGCACATAACAAATATTATGCAGATATAAAGCTTGACACAAATACAGGAAACACTTATATAGATGGTATATATTTTGAGCCAACCCAAA 2368
 |||||||||||||||||||||||||||||| |||||||||||||||||||||||||||||||||||||||||||||||||||||||||||||||||||||||||||||| ||||| ||||
JGS1987 TTAAAGTTCCAAGTGACCAAGAAATACTAGATGCACATAACAAATATTATGCAGATATAAAGCTTGACACAAATACAGGAAACACTTATATAGATGGTATATATTTTGAACCAACTCAAA
PB-1 CTAATAAAGAAGCTCTTGATTATATTCAAAAATATAGAGTTGAAGCAACTTTGCAATATTCAGGATTCAAAGATATTGGAACTAAGGATAAAGAAGTACGTAATTATTTAGGAGATCCAA 2488
 ||||||||||||||||||||||||||||||||||||||||||||||||||||||||||||||||||| ||||||||||||||||||||||||||| ||||||||||||||||||||| ||
JGS1987 CTAATAAAGAAGCTCTTGATTATATTCAAAAATATAGAGTTGAAGCAACTTTGCAATATTCAGGATTTAAAGATATTGGAACTAAGGATAAAGAAATACGTAATTATTTAGGAGATCAAA
PB-1 ATCAACCTAAAACTAATTATATTAATCTTAGAAGTTATTTTACTAGTGGAGAAAATGTTATGACATATAAAAAATTAAGAATATATGCAGTTACACCTGATAATAGAGAATTATTAGTGC 2608
 | |||||||||||||||||||||||| |||||||||||||||||||||||||||||||||||||||||||||||||||||||||||||||||||||||||||||||||| ||||||||||
JGS1987 ACCAACCTAAAACTAATTATATTAATTTTAGAAGTTATTTTACTAGTGGAGAAAATGTTATGACATATAAAAAATTAAGAATATATGCAGTTACACCTGATAATAGAGAGTTATTAGTGC
PB-1 TTAGTGTTAATTAA 2622
 ||||||||||||||
JGS1987 TTAGTGTTAATTAA 5497
Comparison of nucleotide sequence of the variant ibp gene of pCPPB-1 against the ibp gene of classical type E isolates (JGS1987). (Continued)
