# Supplementary material for: Identification of Novel Clostridium perfringens Type E Strains That Carry an Iota Toxin Plasmid with a Functional Enterotoxin Gene
Source: PLoS One. 2011 May 31;6(5):e20376. doi: 10.1371/journal.pone.0020376 (PMC3105049; doi:10.1371/journal.pone.0020376)
Supplement: Figure S6 — Schematic presentation showing putative cpe promoter sequences in the chromosomal cpe strain NCTC10240 versus those in the the PB-1 strain carrying the plasmid borne variant cpe gene. (PPT) [file pone.0020376.s006.ppt]

## Slide 1
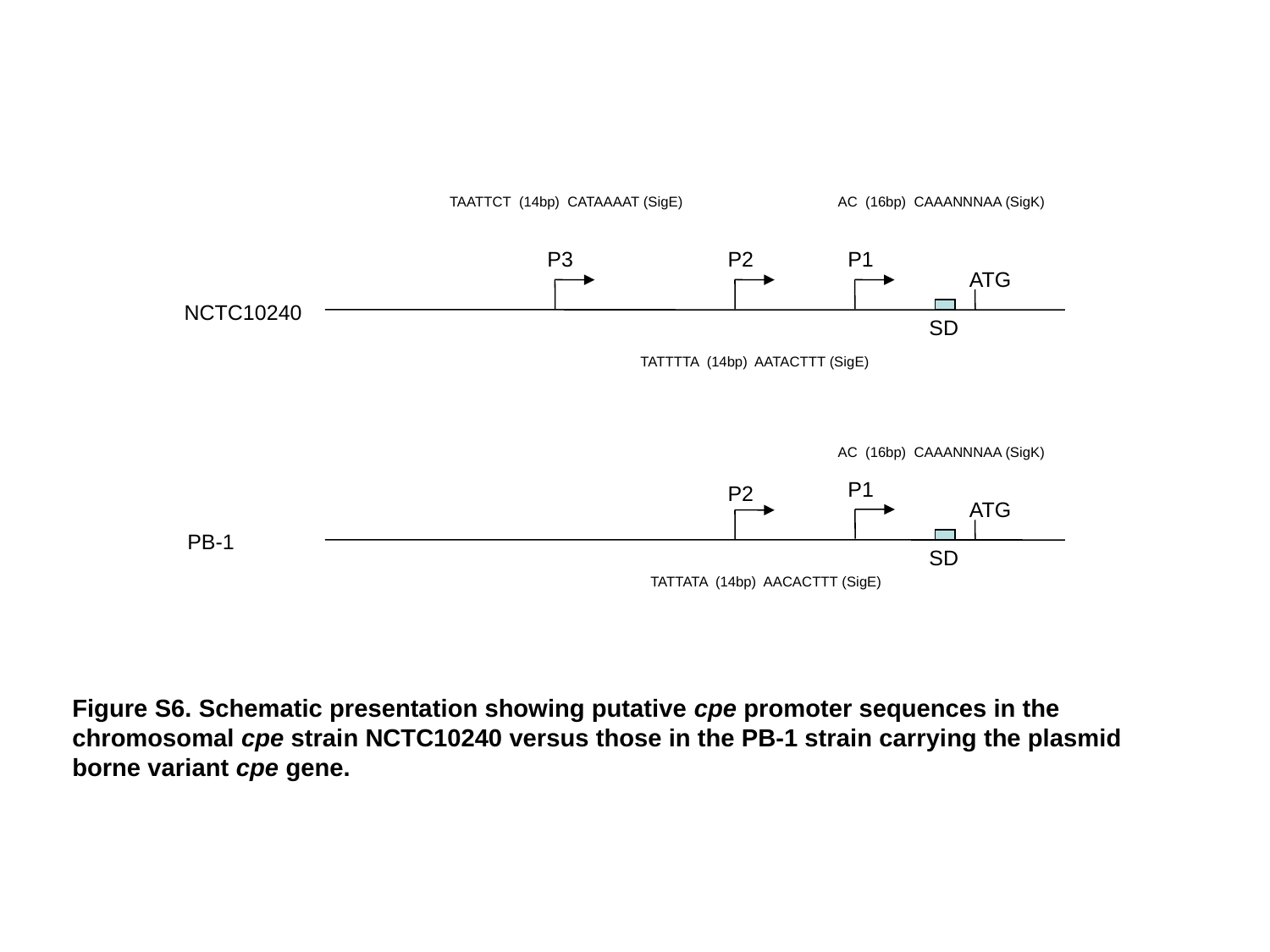

TAATTCT (14bp) CATAAAAT (SigE)
AC (16bp) CAAANNNAA (SigK)
P3
P2
P1
ATG
NCTC10240
SD
TATTTTA (14bp) AATACTTT (SigE)
AC (16bp) CAAANNNAA (SigK)
P1
P2
ATG
PB-1
SD
TATTATA (14bp) AACACTTT (SigE)
Figure S6. Schematic presentation showing putative cpe promoter sequences in the chromosomal cpe strain NCTC10240 versus those in the PB-1 strain carrying the plasmid borne variant cpe gene.
