# Supplementary material for: Identification of Novel Clostridium perfringens Type E Strains That Carry an Iota Toxin Plasmid with a Functional Enterotoxin Gene
Source: PLoS One. 2011 May 31;6(5):e20376. doi: 10.1371/journal.pone.0020376 (PMC3105049; doi:10.1371/journal.pone.0020376)
Supplement: Figure S7 — Overlapping PCR assays of plasmid encoding the variant cpe gene in four isolates using primers designed to amplify the pCPF5603/pCPPB-1 transfer region. Shown are results obtained using DNA from strains (PB-1, 3441, TGII002 and TGII003), which carry the plasmid-borne variant cpe gene or from F4969 and F5603 which carry the classical cpe plasmids pCPF4969 and pCPF5603. Using primers previously described, the region assayed with PCR reaction (T6 to T16) contained the eight tcp (tcpA to tcpI) genes and the intP gene, which genes are thought to be necessary for plasmid transfer (Table S4) [11], [13]. The overlapping PCR (reaction T14t to T16t) used newly constructed primers based on the sequence information of the tcpA region on pCPPB-1 (Table S4). (PPT) [file pone.0020376.s007.ppt]

## Slide 1
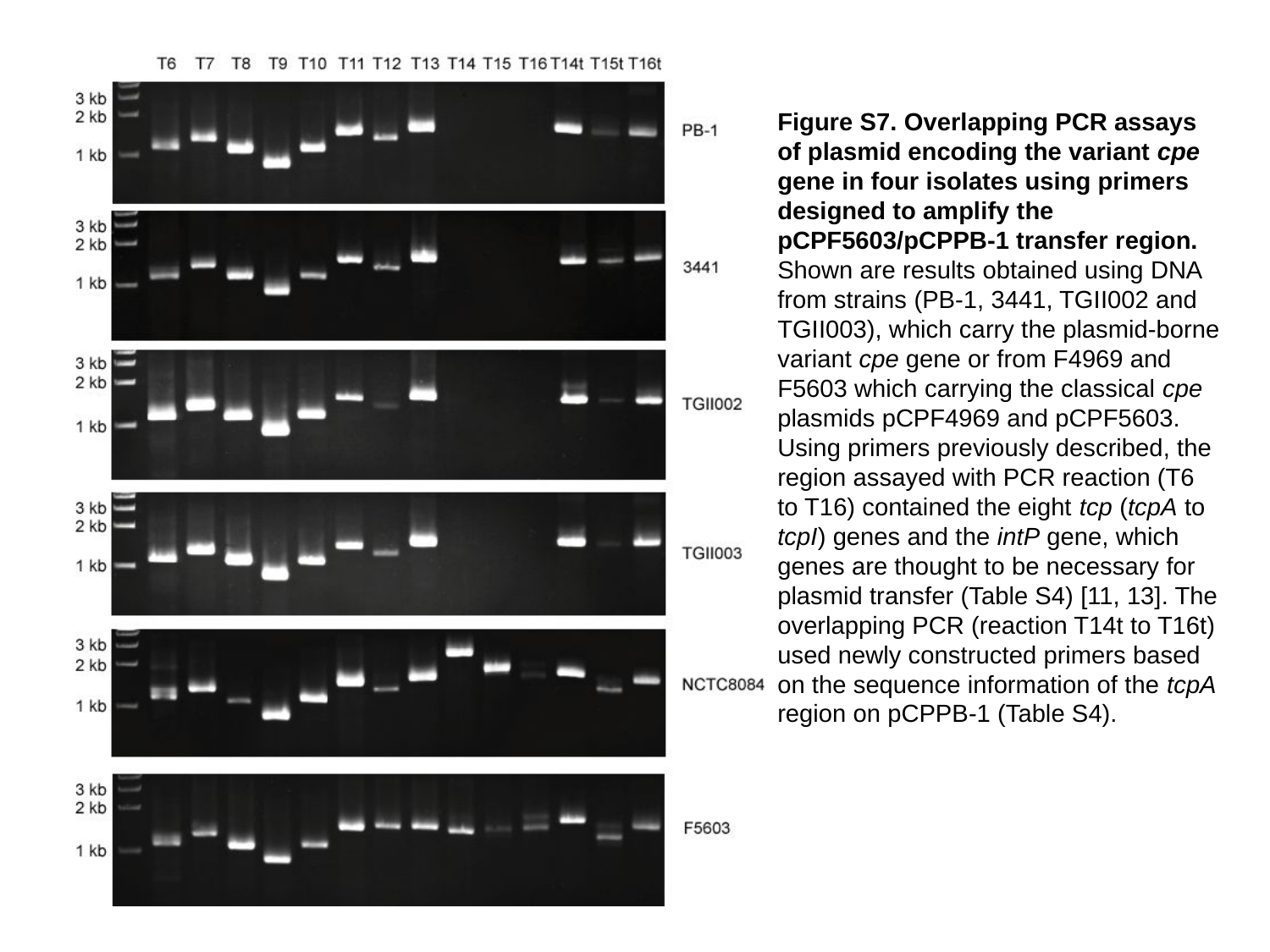

Figure S7. Overlapping PCR assays of plasmid encoding the variant cpe gene in four isolates using primers designed to amplify the pCPF5603/pCPPB-1 transfer region. Shown are results obtained using DNA from strains (PB-1, 3441, TGII002 and TGII003), which carry the plasmid-borne variant cpe gene or from F4969 and F5603 which carrying the classical cpe plasmids pCPF4969 and pCPF5603. Using primers previously described, the region assayed with PCR reaction (T6 to T16) contained the eight tcp (tcpA to tcpI) genes and the intP gene, which genes are thought to be necessary for plasmid transfer (Table S4) [11, 13]. The overlapping PCR (reaction T14t to T16t) used newly constructed primers based on the sequence information of the tcpA region on pCPPB-1 (Table S4).
