# Supplementary material for: Identification of Novel Clostridium perfringens Type E Strains That Carry an Iota Toxin Plasmid with a Functional Enterotoxin Gene
Source: PLoS One. 2011 May 31;6(5):e20376. doi: 10.1371/journal.pone.0020376 (PMC3105049; doi:10.1371/journal.pone.0020376)
Supplement: Figure S8 — Overlapping PCR assays of plasmid carrying the variant cpe gene in four isolates using primers designed to amplify the pCPF4969 variable region. Shown are results using DNA specimens from strains (PB-1, 3441, TGII002 and TGII003), which carries the variant cpe gene or from F4969 and F5603, which are strains carrying the classical cpe plasmid pCPF4969 and pCPF5603 [11]. (PPT) [file pone.0020376.s008.ppt]

## Slide 1
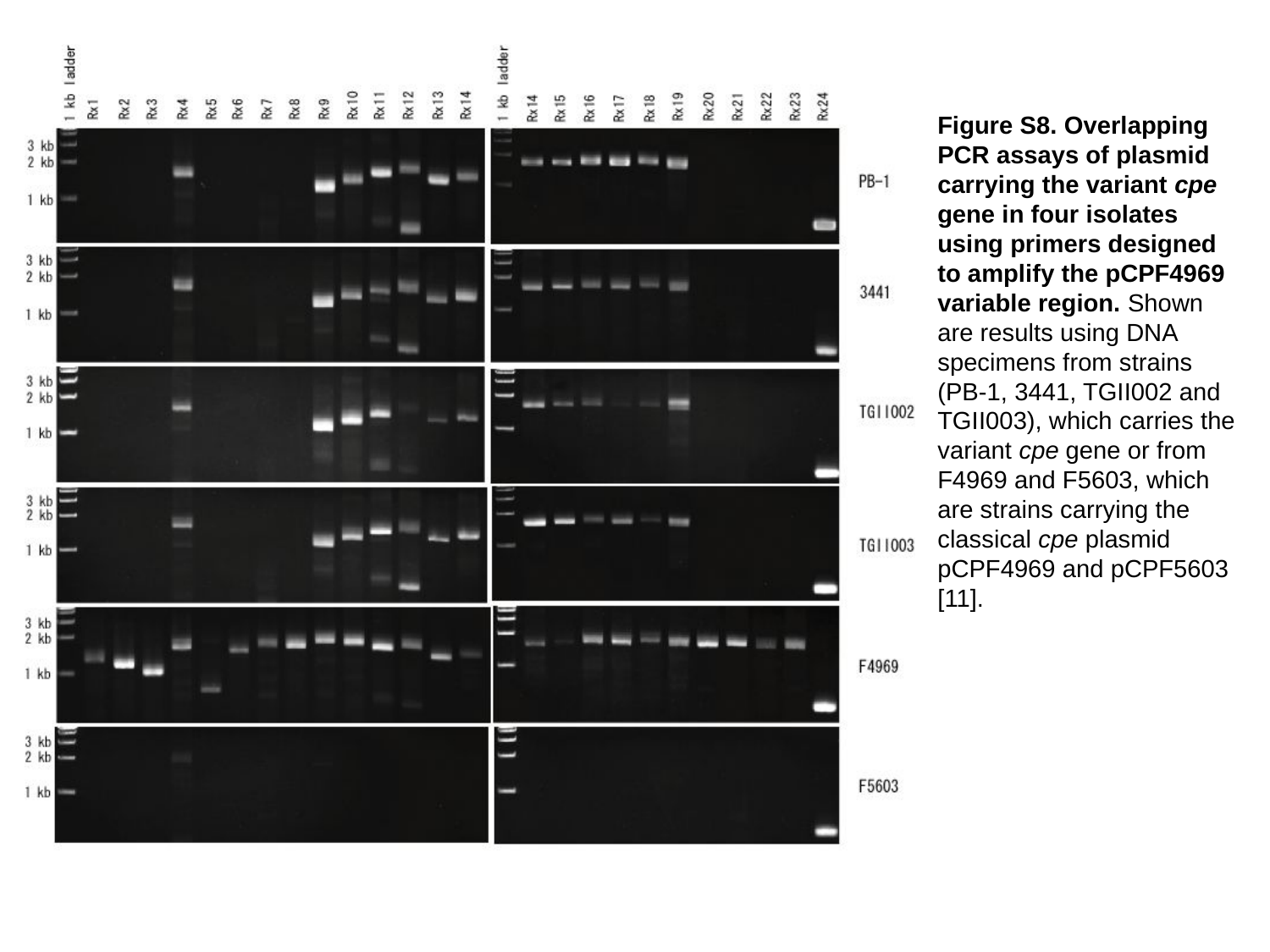

Figure S8. Overlapping PCR assays of plasmid carrying the variant cpe gene in four isolates using primers designed to amplify the pCPF4969 variable region. Shown are results using DNA specimens from strains (PB-1, 3441, TGII002 and TGII003), which carries the variant cpe gene or from F4969 and F5603, which are strains carrying the classical cpe plasmid pCPF4969 and pCPF5603 [11].
