# Supplementary material for: Identification of Novel Clostridium perfringens Type E Strains That Carry an Iota Toxin Plasmid with a Functional Enterotoxin Gene
Source: PLoS One. 2011 May 31;6(5):e20376. doi: 10.1371/journal.pone.0020376 (PMC3105049; doi:10.1371/journal.pone.0020376)
Supplement: Figure S9 — Overlapping PCR assays of the plasmids encoding the variant cpe gene in four isolates using primers designed to amplify the toxin region. Shown are results using DNA from strains (PB-1, 3441, TGII002 and TGII003), which carries the variant cpe gene and variant iota genes, and from NCTC8084 type E strain carrying the silent cpe gene and iota genes. (PPT) [file pone.0020376.s009.ppt]

## Slide 1
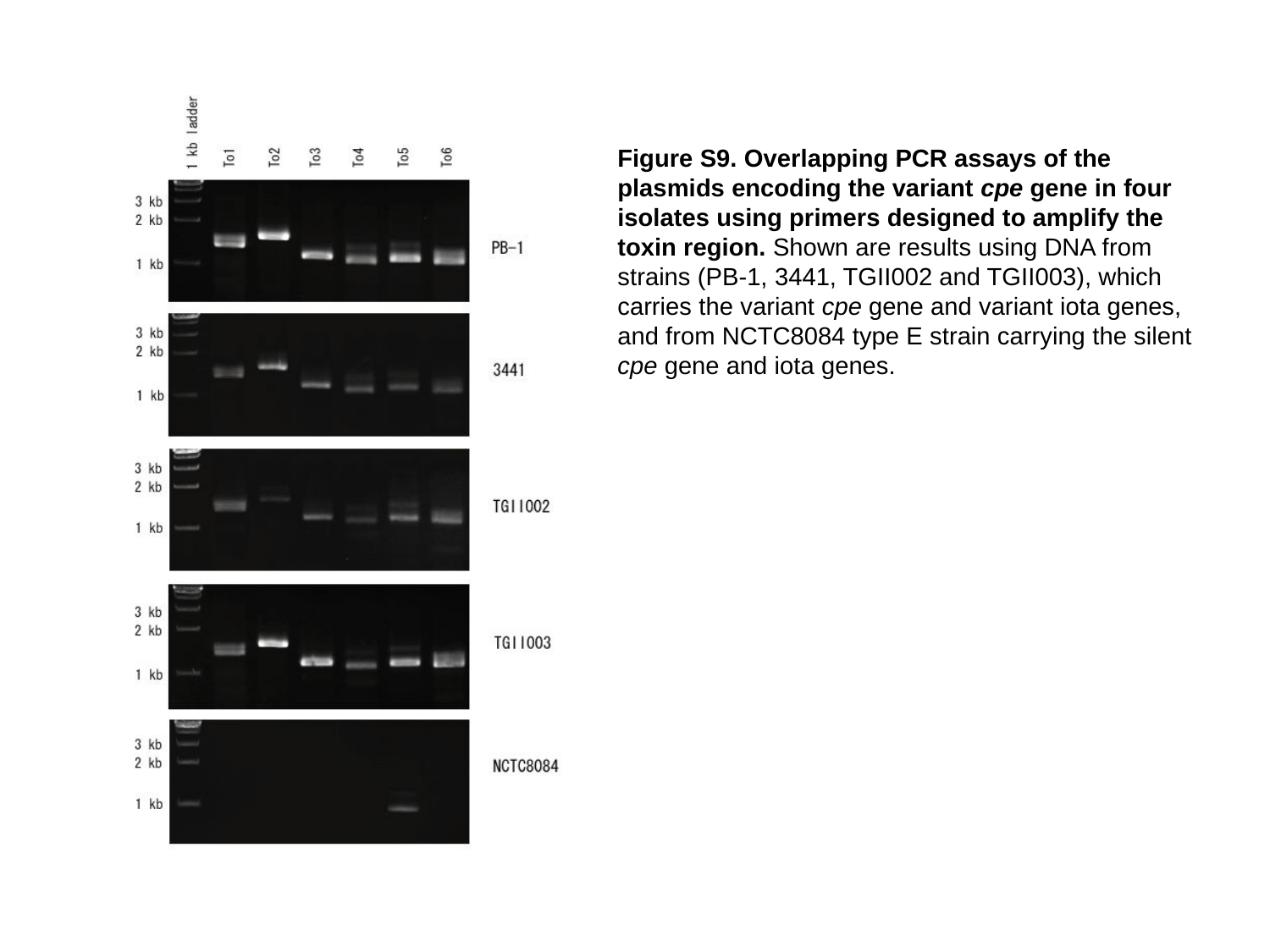

Figure S9. Overlapping PCR assays of the plasmids encoding the variant cpe gene in four isolates using primers designed to amplify the toxin region. Shown are results using DNA from strains (PB-1, 3441, TGII002 and TGII003), which carries the variant cpe gene and variant iota genes, and from NCTC8084 type E strain carrying the silent cpe gene and iota genes.
